# Antioxidant Role for Lipid Droplets in a Stem Cell Niche of *Drosophila*

**DOI:** 10.1016/j.cell.2015.09.020

**Published:** 2015-10-08

**Authors:** Andrew P. Bailey, Grielof Koster, Christelle Guillermier, Elizabeth M.A. Hirst, James I. MacRae, Claude P. Lechene, Anthony D. Postle, Alex P. Gould

**Affiliations:** 1 The Francis Crick Institute, Mill Hill Laboratory, The Ridgeway, Mill Hill, London NW7 1AA, UK; 2 Academic Unit of Clinical & Experimental Sciences, Faculty of Medicine, Sir Henry Wellcome Laboratories, Southampton General Hospital, Southampton SO16 6YD, UK; 3 National Resource for Imaging Mass Spectroscopy, Harvard Medical School and Brigham and Women’s Hospital, Cambridge, MA 02139, USA

## Abstract

Stem cells reside in specialized microenvironments known as niches. During *Drosophila* development, glial cells provide a niche that sustains the proliferation of neural stem cells (neuroblasts) during starvation. We now find that the glial cell niche also preserves neuroblast proliferation under conditions of hypoxia and oxidative stress. Lipid droplets that form in niche glia during oxidative stress limit the levels of reactive oxygen species (ROS) and inhibit the oxidation of polyunsaturated fatty acids (PUFAs). These droplets protect glia and also neuroblasts from peroxidation chain reactions that can damage many types of macromolecules. The underlying antioxidant mechanism involves diverting PUFAs, including diet-derived linoleic acid, away from membranes to the core of lipid droplets, where they are less vulnerable to peroxidation. This study reveals an antioxidant role for lipid droplets that could be relevant in many different biological contexts.

## Highlights

• Oxidative stress stimulates lipid droplet biosynthesis in a neural stem cell niche
• Lipid droplets protect niche and neural stem cells from damaging PUFA peroxidation
• PUFAs are less vulnerable to peroxidation in lipid droplets than in cell membranes

## Graphical Abstract

**Figure:**
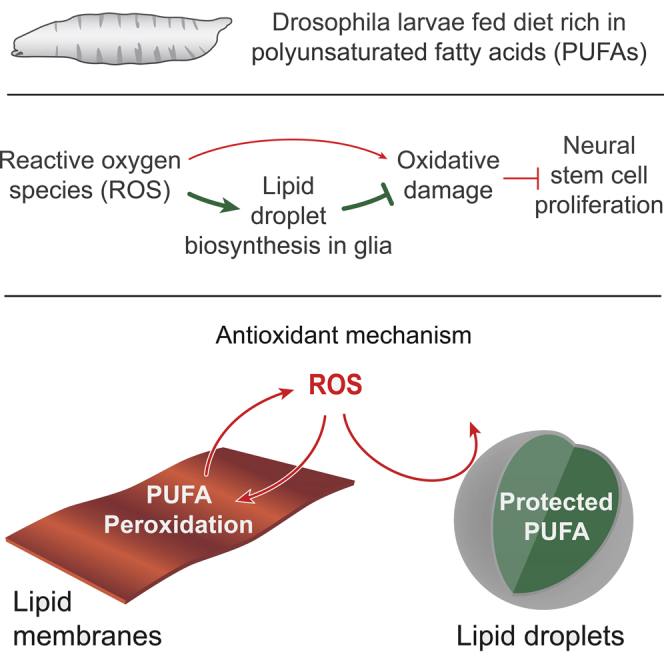

## Teaser

Lipid droplets can act as antioxidant organelles that protect *Drosophila* neural stem cells from hypoxia-triggered ROS. They sequester polyunsaturated fatty acids away from plasma membranes, thereby shielding these lipids from harmful peroxidation that would otherwise compromise proliferation.

## Introduction

Stem and progenitor cells drive growth during development, tissue regeneration, and tumorigenesis (Blanpain and Fuchs, 2014; Hanahan and Weinberg, 2011; Slack, 2008). They are regulated by growth factors and other signals from a local microenvironment called the niche (Scadden, 2006). In many different physiological contexts, tissues containing stem cells are exposed to low levels of dietary nutrients or oxygen (Barker, 1995; Dunwoodie, 2009; Pugh and Ratcliffe, 2003). Multiple pathways sense these environmental stresses and trigger responses that can significantly impact upon metabolism (Tower, 2012). Stem, progenitor, and tumor cells tend to utilize proportionately more glycolysis and less oxidative phosphorylation than differentiated cells (Burgess et al., 2014). The low oxygen consumption of glycolytic metabolism appears well matched to the physiological hypoxia of the niche in which many different stem cells reside (Burgess et al., 2014; Mohyeldin et al., 2010). This hypoxic microenvironment can itself play a key role in regulating the balance between stem cell quiescence, self-renewal, and differentiation. Hypoxic stem cells and their niche are often associated with hypoxia inducible factor (HIF) activity and also with an increase in reactive oxygen species (ROS), both of which can act as signals promoting glycolysis and metabolic reprograming (Lee and Simon, 2012; Ushio-Fukai and Rehman, 2014). If hypoxia or other forms of oxidative stress induce ROS levels that are high enough to exceed cellular defense mechanisms, then they promote harmful oxidation and peroxidation chain reactions that can damage lipids, proteins, and nucleic acids (Negre-Salvayre et al., 2008). Many types of stem cells therefore synthesize high levels of antioxidants such as glutathione (GSH) and also antioxidant enzymes such as superoxide dismutase (SOD) and catalase (Cat) in order to defend themselves against ROS (Wang et al., 2013).

Neural stem cells are critical for growth of the mammalian CNS during development and also for neuronal turnover in the subventricular zone and dentate gyrus during adulthood (Okano and Temple, 2009; Taverna et al., 2014). In common with other stem cells, they are known to reside in a niche that is hypoxic even when the external environment is normoxic and nutrient rich (Cunningham et al., 2012). It is also well described that neonatal brain size is highly protected or spared from the increased hypoxia and malnutrition that are experienced during intrauterine growth restriction (Barker, 1995; Gruenwald, 1963). How then do these environmental stresses alter the properties of neural stem cells and/or their niche and which, if any, of these changes are adaptive for brain sparing?

The developing *Drosophila* CNS is a useful model for investigating the effects of environmental stresses upon neural stem and progenitor cells. Embryonic and larval neuroepithelia give rise to neural stem cells called neuroblasts, which divide asymmetrically to generate multiple types of neurons and glia (Homem and Knoblich, 2012; Pearson and Doe, 2004; Skeath and Thor, 2003). As with other growing and dividing *Drosophila* cells, larval neuroblasts rely heavily upon glycolytic metabolism (Grant et al., 2010; Homem et al., 2014; Tennessen et al., 2011). Properties of neuroblasts such as their type, number of divisions, and lineage composition differ from region to region within the CNS (Li et al., 2013; Sousa-Nunes et al., 2010). In the central brain and ventral ganglion, neuroblasts undergo two periods of neurogenesis separated by a period of cell-cycle arrest called quiescence. Exit from quiescence (reactivation) occurs during early larval stages and requires dietary amino acids (Britton and Edgar, 1998; Truman and Bate, 1988). Amino acids are sensed by the target of rapamycin (TOR) pathway in the fat body and activate a systemic relay signal that triggers insulin-like peptide (Ilp) expression in glia, an important niche for larval neuroblasts (Chell and Brand, 2010; Colombani et al., 2003; Dumstrei et al., 2003; Sousa-Nunes et al., 2011; Spéder and Brand, 2014). Glial Ilps then activate the insulin-like receptor (InR) in neuroblasts leading to TOR and phosphatidylinositol 3-kinase (PI3K) signaling and re-entry into the cell cycle (Chell and Brand, 2010; Sousa-Nunes et al., 2011). Later during development, the growth of neuroblast lineages becomes largely independent of all dietary nutrients, providing a model for brain sparing (Cheng et al., 2011). At this stage, niche glia express a secreted growth factor, jelly belly (Jeb), regardless of whether dietary nutrients are present or absent. Jeb then activates its receptor anaplastic lymphoma kinase (Alk) in neuroblasts, thus promoting constitutive rather than nutrient-dependent PI3K signaling and growth (Cheng et al., 2011). Similar brain sparing specific to older larvae has also been identified in the *Drosophila* visual system, where the late asymmetric but not the early symmetric divisions of neural progenitors tend to be spared from nutrient restriction (Lanet et al., 2013). Importantly, however, it has not been clear in any region of the *Drosophila* CNS whether neuroblasts are also protected against other environmental stresses such as hypoxia. We now investigate this issue using imaging mass spectrometry, CNS lipidomics, and genetic manipulations specific for the glial niche or the neuroblast lineage. This combined approach identifies a molecular mechanism by which lipid metabolism in a stem cell niche can protect the stem cells themselves from oxidative damage.

## Results

### The Proliferation of Neuroblast Lineages Is Spared during Hypoxia

**Figure 1:**
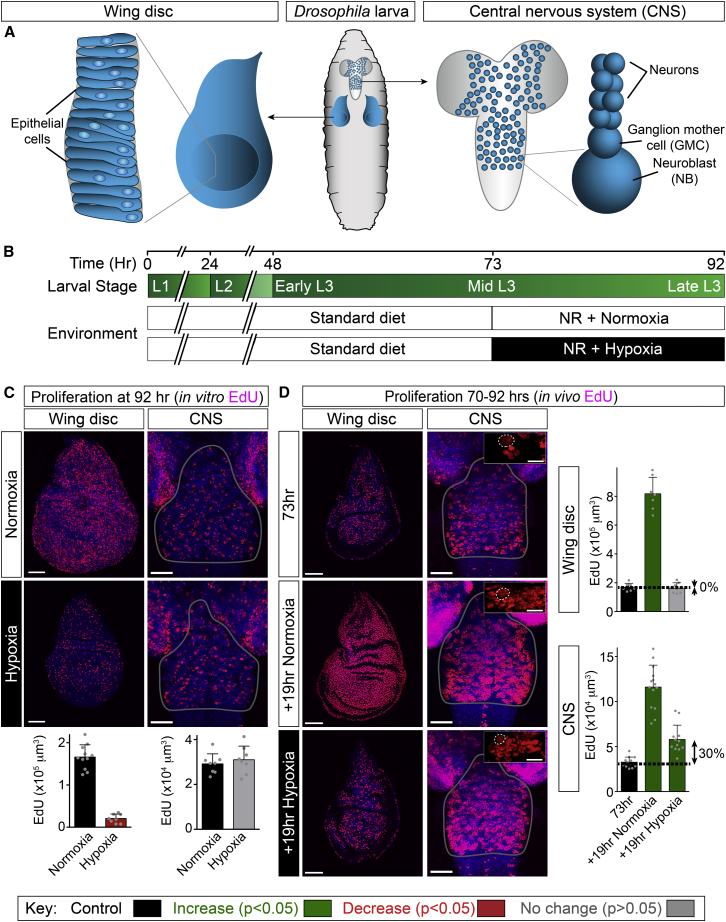
Neuroblast Proliferation Is Spared during Environmental Hypoxia (A) Cartoon of *Drosophila* larva, showing the developing wing discs and CNS. Most proliferating cells correspond to neuroblasts (NBs) and ganglion mother cells (GMCs) within the CNS and to epithelial progenitors within the wing disc. (B) Larval development timeline (hours after larval hatching), depicting the three larval instars and the hypoxia (2.5% oxygen) regimen used. (C) In vitro EdU assay shows that cell proliferation in neuroblast lineages (CNS) is more resistant to hypoxia than that of epithelial progenitors (wing disc). After 22 hr of normoxic or hypoxic NR, tissues were dissected and incubated in vitro with EdU for 1 hr. (D) In vivo EdU assay indicates that cell proliferation in neuroblast lineages (CNS) is more resistant to hypoxia than that of epithelial progenitors (wing disc). The CNS and wing disc are shown at 73 hr (after 3 hr of normoxic NR with EdU) and also at 92 hr (after a further 19 hr of normoxic or hypoxic NR in the presence of EdU). CNS insets in (D) show high-magnification views of EdU^+^ cells, large neuroblasts (dotted circle) are already EdU^+^ at 73 hr and contribute to the final EdU volumes. Histograms in (C) and (D) depict the average volume of EdU^+^ cells incorporated per wing disc or per thoracic CNS. Scale bars, 50 μm, inset scale bars, 10 μm. In this and subsequent figures, histograms show the mean, scatterplots show individual data points, and error bars are 1 SD. The key shows that histogram bars are colored according to t tests (p < 0.05): significant decrease (red), significant increase (green), or no significant change (gray) from the control (black). See also Figure S1.

*Drosophila* larvae develop into undersized yet viable adults when starved during the third instar, (Bakker, 1959; Beadle et al., 1938). We previously used a nutrient restriction (NR) protocol in which third instar larvae are transferred from a yeast/glucose/cornmeal to an agarose medium (Cheng et al., 2011). During NR, there is no increase in overall body mass but growth and cell division are spared in CNS neuroblast lineages and also, to a lesser extent, in epithelial progenitors of the wing disc (Cheng et al., 2011; Figure 1A).

**Figure S1:**
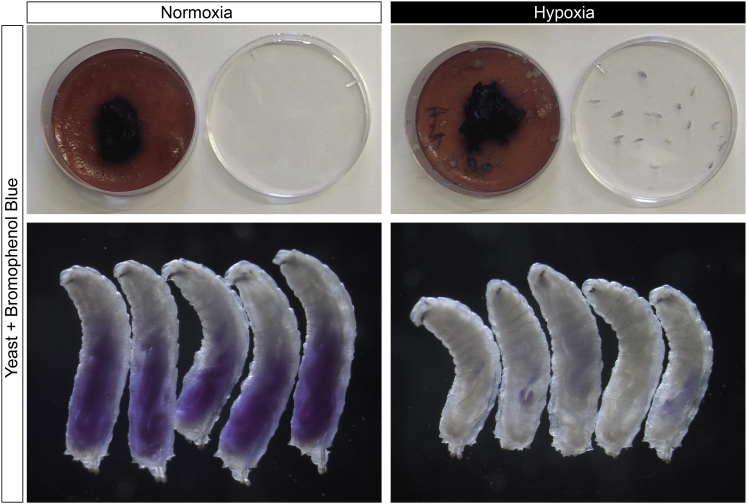
Hypoxia Inhibits Larval Food Intake, Related to Figure 1 Top panels show petri dishes containing colored food (yeast paste containing bromophenol blue) and their corresponding lids. Petri dishes contained mid-third instar larvae subject to normoxia (20.9% oxygen, left panels) or hypoxia (2.5% oxygen, right panels) for 2.5 hr. Bottom panels show high magnification views of larvae exposed to normoxia (left panels) or hypoxia (right panels) at the end of the experiments. Under normoxia, larvae remain in the food and ingested food is clearly visible in the gut. In contrast, hypoxia induces larvae to crawl out of the food and to ingest very little.

We first quantified the effects of hypoxia upon cell proliferation using the thymidine analog 5-ethynyl-2′-deoxyuridine (EdU). This method measures the proliferation of neuroblast lineages, with glia representing less than 6% of total EdU^+^ cells (Sousa-Nunes et al., 2011; data not shown). Larvae exposed to hypoxia are known to crawl away from yeast paste (Wingrove and O’Farrell, 1999), and we found that, in a 2.5% oxygen environment, they do not ingest significant quantities of food (Figure S1). This behavioral response may normally serve to prevent anoxia by limiting the duration of burrowing bouts in semi-liquid medium, and it can be synchronized by applying artificial hypoxic-normoxic cycles (Movie S1). To assess specifically the effects of low oxygen, rather than reduced feeding, we raised larvae on yeast/glucose/cornmeal until mid-third instar (∼70 hr after larval hatching, ALH) and then transferred them to NR medium either in normoxia or hypoxia (Figure 1B). EdU incorporation by thoracic neuroblast lineages and by wing disc progenitors was measured at the end (1 hr in vitro) or throughout (22 hr in vivo) the hypoxic period (Figures 1C and 1D). For both tissues, the percentage of cell proliferation spared in hypoxia (relative to normoxia) is smaller with the in vivo cumulative than with the in vitro endpoint assay. This difference may reflect brief exposure of hypoxic larvae to normoxia prior to dissection and in vitro EdU incubation. Importantly, however, both methodologies clearly demonstrate that cell divisions of neuroblasts are more resistant to hypoxia than those of progenitors in the developing wing.

### Hypoxia Induces Lipid Droplets in Subperineurial and Cortex Glia

**Figure 2:**
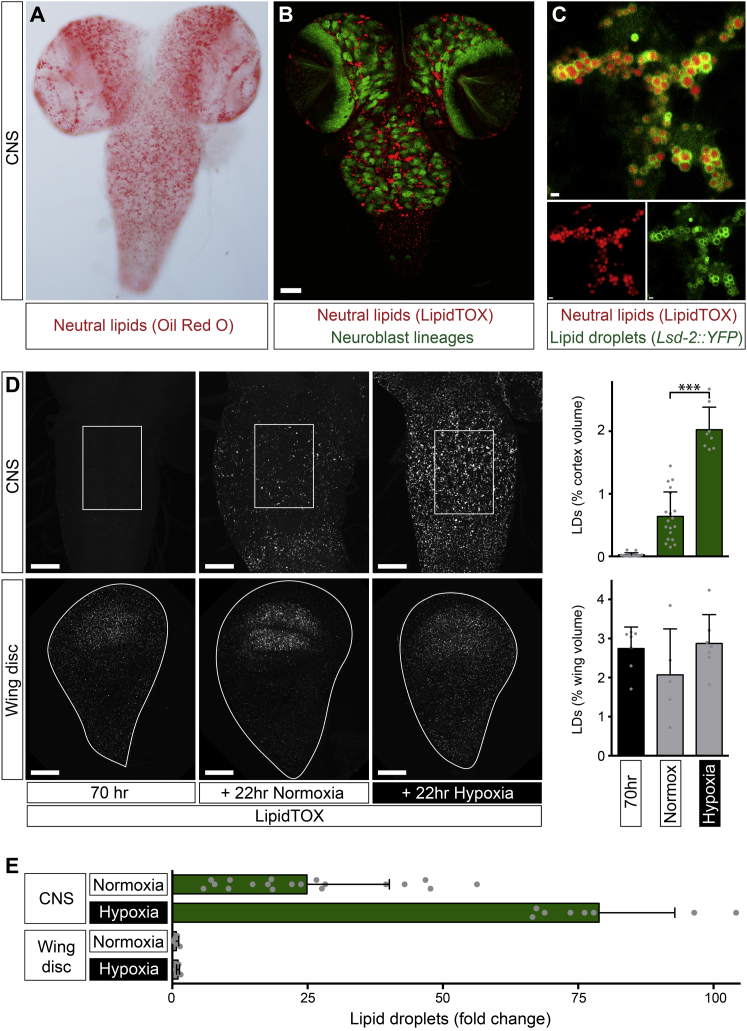
Hypoxia Induces Lipid Droplets in the CNS (A) Oil red O-stained neutral lipids in the CNS of a late-third instar larva (control genotype: *w*^*1118*^) raised on a standard diet. (B) LipidTOX stained neutral lipids in the CNS of a late-third instar larva raised on a standard diet. Neutral lipids (red) are prominent in the central brain and ventral nerve cord but do not overlap with neuroblast lineages (green: *nab-Gal4 > CD8::GFP*). Scale bar, 50 μm. (C) Neutral lipids (LipidTOX, red) in the thoracic ganglion of the late third instar CNS accumulate in clusters of lipid droplets labeled with *Lsd-2::YFP* (green). Scale bars, 1 μm. (D) Lipid droplets (LipidTOX) in the CNS of a mid-third instar (70 hr) larva fed on a standard diet increase during NR normoxia and become more numerous during NR hypoxia. The lipid droplet (LD) content of the wing disc does not change significantly during hypoxia. It is expressed as percentage of volume within the thoracic region of interest of the ventral nerve cord (white boxes, upper row) or the entire wing disc (white outlines, lower row). Scale bars, 50 μm. (E) Lipid droplets increase ∼25-fold in normoxia and ∼80-fold in hypoxia in the CNS but do not significantly change in the wing disc. Note that fold change values in this figure are normalized to the 70 hr start points of Figure 2D. See also Figure S2.

**Figure S2:**
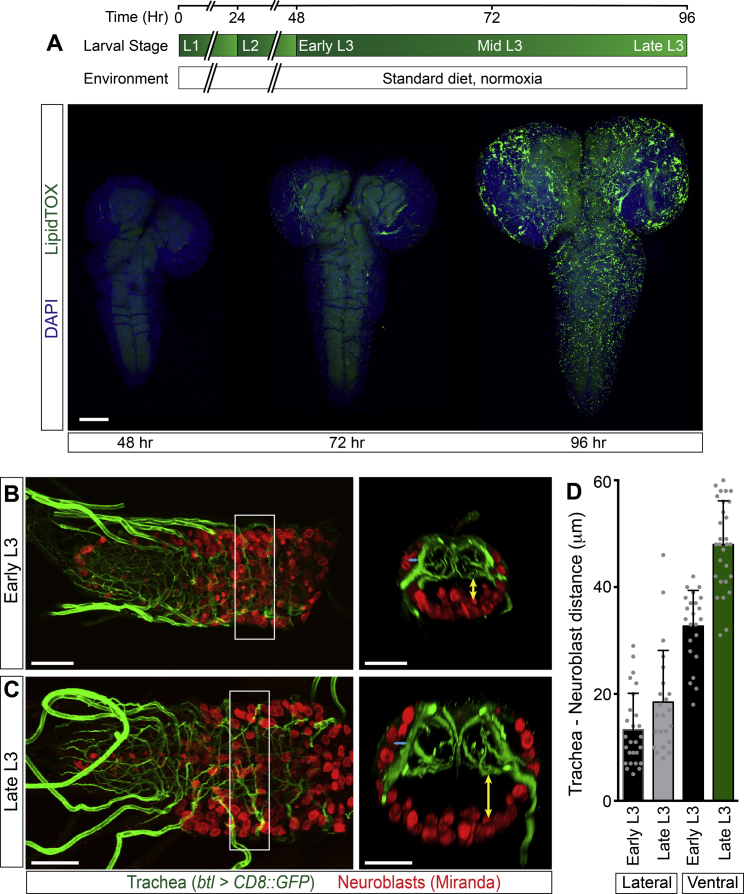
CNS Lipid Droplets Accumulate and the Neuroblast-Tracheal Distance Increases during Normal Development, Related to Figure 2 (A) LipidTOX staining of the CNS at early (48 hr), mid (72 hr) or late (96 hr) third instar showing a progressive lipid droplet accumulation during development. Larvae were raised on standard diet in ambient normoxia. Scale bar: 50 μm. (B and C) The CNS tracheal network and neuroblasts are shown at early (B: 48 hr) and late (C: 96 hr) third instar. Tracheal-specific GFP expression (*btl > CD8::GFP*, green) and the neuroblast marker Miranda (red) are shown. The main tracheal network is in the neuropil, whereas neuroblasts are in the cortex. Scale bars: 50 μm. (D) The tracheal-neuroblast distance, measured from confocal z-plane reconstructions of the ventral nerve cord (white boxes in B and C). Between early and late third instar, a significant ∼1.5 fold increase is observed in the average distance (yellow arrows) between ventral neuroblasts and the tracheal network, although there is no significant change in lateral regions (blue arrows).

We next investigated the metabolic specializations underlying the striking hypoxia resistance of neuroblast lineages. Highly active glycolysis in dividing neuroblasts is unlikely to account per se for their selective hypoxia resistance as it is also present in wing discs (de la Cova et al., 2014; Homem et al., 2014; data not shown). However, using neutral lipid stains (oil red O and LipidTOX), we were intrigued to observe lipid droplets in the developing CNS (Figures 2A and 2B). Lipid droplets are cytoplasmic organelles comprising a core of neutral lipids, such as triacylglycerols (TAGs) and cholesteryl esters, surrounded by a phospholipid monolayer associated with lipases and other proteins regulating droplet biogenesis, lipid storage, and release (Kühnlein, 2012; Walther and Farese, 2012; Young and Zechner, 2013). In *Drosophila*, lipid droplets in adipose tissue (known as fat body) express the regulatory protein Lsd-2 (perilipin-2) on their surface (Bickel et al., 2009; Kühnlein, 2011), and we find that this is also the case for droplets in the CNS (Figure 2C). CNS lipid droplets, however, tend to be 1–2 μm diameter, smaller than many of those in the fat body. On standard food in ambient normoxia, the number of CNS lipid droplets increases during larval development (Figure S2A). This is consistent with a previous lipidomic analysis showing that the late third instar CNS contains significant quantities of TAGs (Carvalho et al., 2012). Lipid droplets in the CNS increase rather than decrease during NR (with normoxia) and so, in contrast to those in fat body, do not appear to correspond to a nutrient store that is depleted during starvation (Figure 2D; Gutierrez et al., 2007). However, lipid droplets increase during development with a concomitant increase in the distance separating neuroblasts from the oxygen-supplying cells of the tracheal network (Figures S2B–S2D). We then tested directly whether low oxygen tension can induce CNS lipid droplets. Strikingly, exposing larvae to hypoxia induces a 3-fold gain in lipid droplets, over and above the normoxic developmental increase (Figures 2D and 2E). Importantly, this strong induction is CNS specific, as lipid droplets in the wing disc (Fauny et al., 2005; Parra-Peralbo and Culi, 2011) do not increase significantly during hypoxia (Figures 2D and 2E). Hence, lipid droplets accumulate in the CNS during normal development and can also be strongly induced by hypoxia.

**Figure 3:**
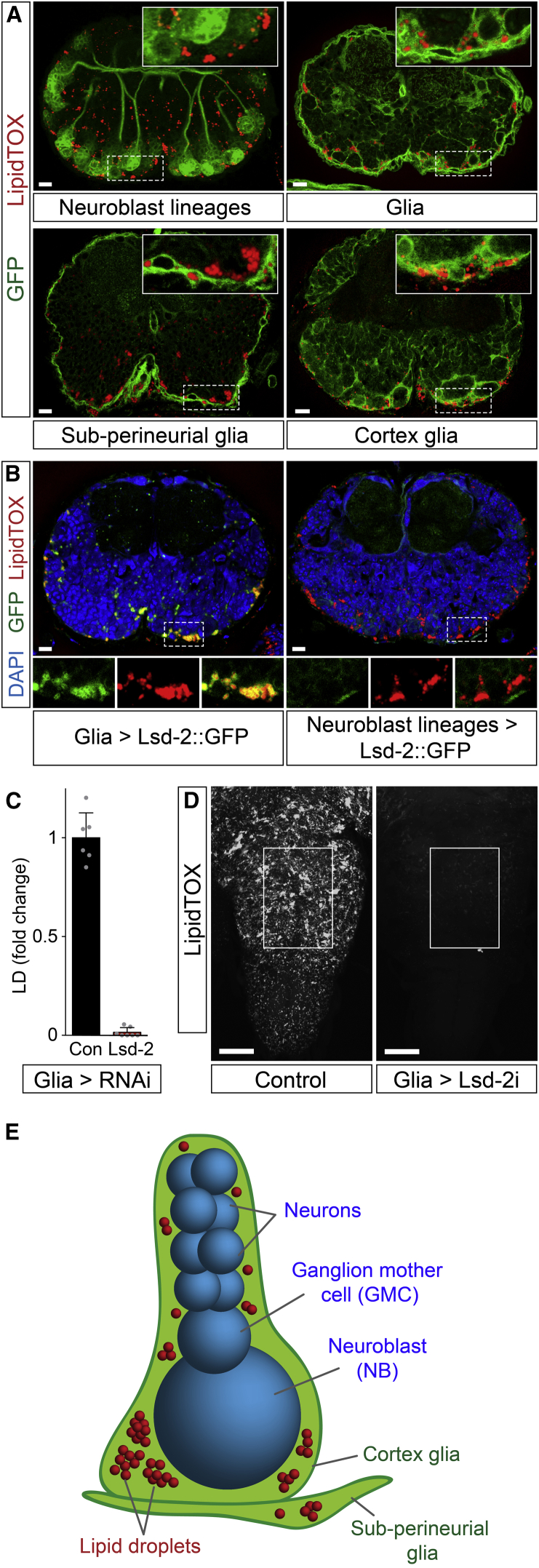
Lipid Droplets Accumulate in Cortex and Subperineurial Glia (A) Transverse sections (dorsal up) of the thoracic CNS from late-third instar larvae raised on standard diet. Lipid droplets (LipidTOX) are observed nearby, but not within neuroblast lineages (labeled with *nab > CD8::GFP*). They localize to glial cells (*repo > CD8::GFP*) including the cortex glia (*nrt*^*GMR37H03*^*> CD8::GFP*) and the subperineurial glia (*moody > CD8::GFP*). Scale bars, 10 μm. (B) Lipid droplets (LipidTOX) localize to glia (*repo > Lsd-2::GFP*) but not to neuroblast lineages (*elav*^*C155*^*> Lsd2::GFP*). Strong Lsd2::GFP (GFP) signal localizes to the surface of glial lipid droplets but low-intensity signal in glia and neuroblast lineages may indicate localization to other organelles such as the ER (Fauny et al., 2005). Scale bars, 10 μm. (C and D) Glial-specific knockdown of Lsd-2 (*repo > Lsd-2 RNAi*) inhibits lipid droplet accumulation in late-third instar larvae raised on standard diet (control: *repo > w*^*1118*^). Scale bars, 50 μm. (E) Diagram showing the distribution of lipid droplets (red) within cortex and subperineurial glia (green) surrounding a neuroblast lineage (blue), comprising a single neuroblast, a ganglion mother cell, and multiple neuronal progeny.

A panel of GFP and Lsd-2::GFP reporters revealed that lipid droplets are primarily found in glial cells but not in neuroblasts or in their neuronal progeny (Figures 3A and 3B). Lsd-2 localizes to the phospholipid monolayer of the lipid droplet surface where it promotes neutral lipid storage by inhibiting TAG lipolysis (Kühnlein, 2011). Specific knockdown in glia (*repo > Lsd-2 RNAi*) prevents the accumulation of CNS lipid droplets during normal fed development, thus confirming their glial localization (Figures 3C and 3D). Reporters specific for glial subtypes showed that the majority of lipid droplet-containing cells correspond to the subperineurial glia of the blood-brain barrier and the cortex glia that enwrap neuroblast lineages (Figures 3A and 3E; Kis et al., 2015). We conclude that CNS lipid droplets mostly localize to the niche (glia) but not to the neural stem cells (neuroblasts).

### Glial Lipid Droplets Sustain Neuroblast Proliferation during Hypoxia

**Figure 4:**
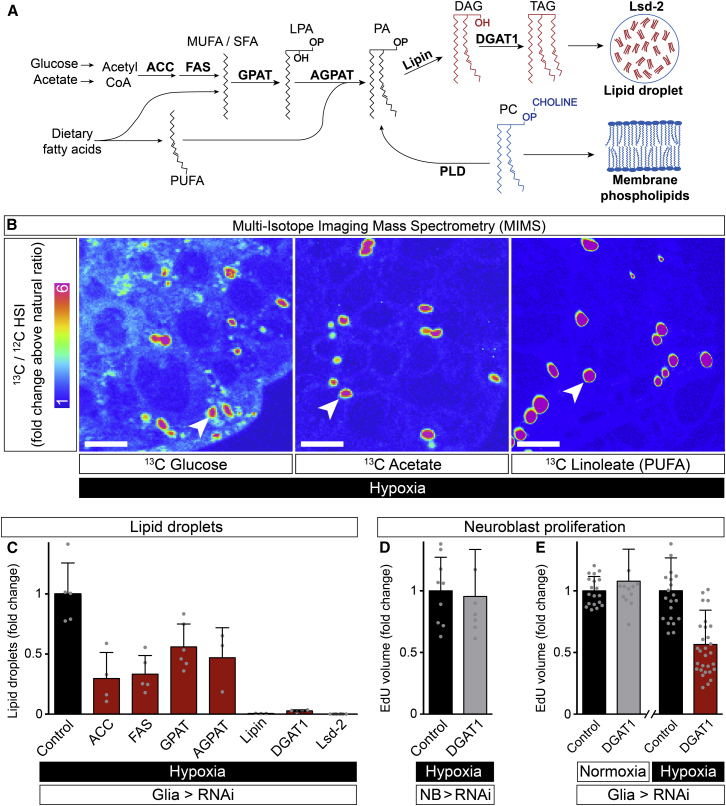
Neuroblast Proliferation during Hypoxia Requires Glial TAG Synthesis (A) TAG synthetic pathway showing the enzymes (bold, see text for details) knocked down by cell-type-specific RNAi in this study. Dietary fatty acids that are saturated (SFA), monounsaturated (MUFA), or polyunsaturated (PUFA) can be incorporated into the pathway at multiple steps, but, for clarity, single entry points are depicted. (B) Multi-isotope imaging mass spectrometry (MIMS) of glial lipid droplets induced by hypoxia in larvae raised on diets containing 1-^13^C-glucose, 1-^13^C acetate, or ^13^C-U-linoleate (PUFA). Hue saturation intensity (HSI) images show that high ^13^C/^12^C ratios are selectively detected in CNS lipid droplets (arrowhead shows one example). Scale bars, 5 μm. (C) Quantification of hypoxia-induced lipid droplets (fold change at endpoint versus *repo > w*^*1118*^ control) with glial-specific gene knockdowns (*repo>RNAi*) of TAG synthetic genes. (D and E) Quantification of neuroblast proliferation (in vivo EdU assays), during normoxia or hypoxia with neuroblast lineage (*nab-Gal4*) or glial (*repo-Gal4*) -specific DGAT1 knockdown (*> DGAT1 RNAi*, control: *> w*^*1118*^). Pooled data are represented as mean ± SD. See also Figure S3.

**Figure S3:**
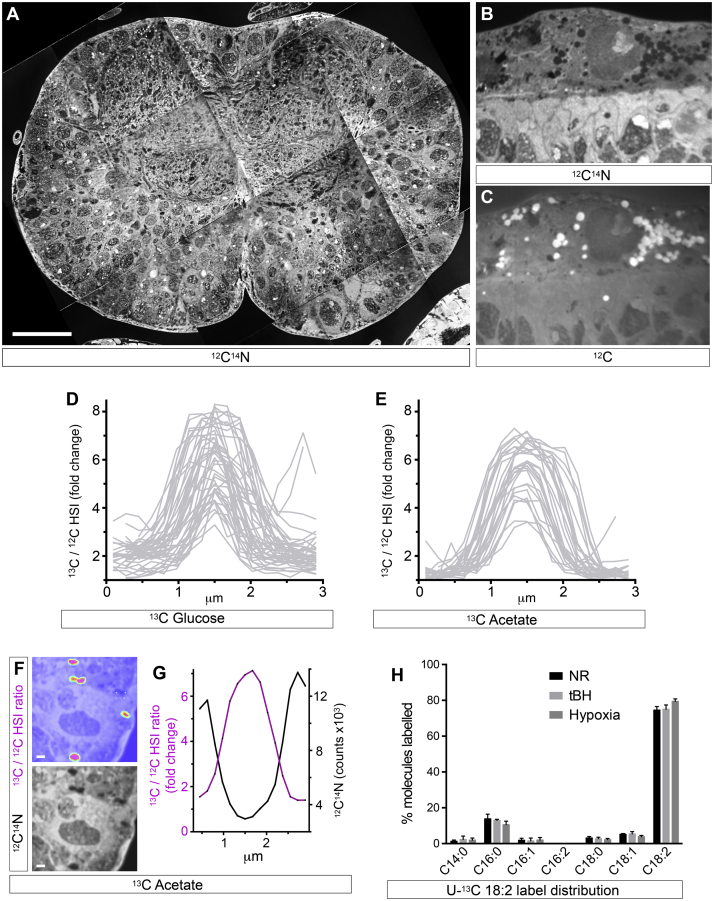
Metabolic Labeling of Glial Lipid Droplets Using MIMS, Related to Figure 4 (A) Stitched composite transverse cross section of the thoracic region of an unlabeled CNS (dorsal up) visualized with multi-isotope imaging mass spectrometry (MIMS) at mass 26 (^12^C^14^N-) to reveal morphology. Scale bar: 50 μm. (B and C) Cross section of the optic lobe of an unlabeled CNS. Images show a single superficial glial cell containing multiple lipid droplets, recognized by characteristic signals of low nitrogen (B, ^12^C^14^N- channel) and high carbon (C, ^12^C channel). (D and E) Quantitations of Hue Saturation Intensity (HSI) images of sectioned lipid droplet profiles. Graphs show ^13^C/^12^C ratio (fold change above background) versus distance (μm) for individual droplet profiles incorporating dietary ^13^C-glucose (D) or ^13^C-acetate (E). MIMS analysis of many lipid droplets gives diameters of 1-2 μm, in agreement with confocal analysis, but apparently smaller droplets likely correspond to ‘glancing’ cross sections that include only the periphery of a droplet. (F) Corresponding ^13^C/^12^C ratio HSI and ^12^C^14^N images of ^13^C-acetate labeled lipid droplets. Scale bars: 1 μm. (G) An example of the ^13^C/^12^C ratio (fold change above background) versus distance (μm) profile for an individual lipid droplet with characteristic low nitrogen content (^12^C^14^N) and a diameter of ∼1.5 μm. (H) GC-MS FAME analysis of major CNS fatty acids incorporating ^13^C following metabolic labeling with dietary U-^13^C-linoleic acid. After NR, hypoxia or tBH (100 mM) stress, ∼80% of ^13^C labeled fatty acids correspond to linoleate (C18:2, n = 3 for each condition, error bars show s.d.). Thus, in the corresponding MIMS analysis (Figure 4B), the majority of ^13^C label remains as linoleate not other fatty acids.

We next determined the metabolic origin of glial lipid droplets using multi-isotope imaging mass spectrometry (MIMS, Steinhauser et al., 2012). Larvae were raised until mid-third instar on diets containing ^13^C-labeled glucose or acetate, major carbon sources for lipogenesis (Figure 4A). Glial lipid droplets were then induced with 22 hr hypoxia. MIMS analysis revealed strong ^13^C incorporation from labeled dietary glucose or acetate into the core of hypoxic lipid droplets (Figures 4B and S3A–S3C). Maximal ^13^C/^12^C ratios in tissue cross sections were observed with lipid droplets of 1–2 μm diameter, consistent with the previous size estimates from confocal microscopy (Figures S3D–S3G). The MIMS analysis demonstrates that de novo fatty acid synthesis contributes neutral lipid cargo to glial lipid droplets. To distinguish whether the relevant de novo lipogenesis is within the glia themselves or in another tissue, glial-specific RNAi was used to knock down six enzymes of TAG biosynthesis (Figure 4A). Lipid droplet induction after 22 hr hypoxia was roughly halved with the knockdown of acetyl-CoA carboxylase (ACC) or a predicted fatty acid synthase (CG3524) (Figure 4C). Fatty acid synthesis is therefore required in a cell-autonomous manner for maximal induction of glial lipid droplets. Given that induction was not completely blocked by knocking down fatty acid synthetic enzymes, we also tested whether there is an additional contribution from dietary fatty acids. Linoleic acid (C18:2) is an omega-6 polyunsaturated fatty acid (PUFA) that is derived from the diet and cannot be synthesized by *Drosophila* (Figure 4A). It is one of the major fatty acids in our standard *Drosophila* diet and remains largely intact following ingestion. Thus, larvae raised on a diet containing ^13^C-linoleic acid retain ∼80% of the ^13^C label incorporated into CNS fatty acids as linoleate (Figure S3H). MIMS analysis of these larvae revealed strong ^13^C incorporation in hypoxia-induced CNS lipid droplets (Figure 4B). Together with the previous results, this indicates that dietary uptake of fatty acids (such as linoleate) and fatty acid synthesis both contribute to glial lipid droplets.

Partial decreases in hypoxia-induced lipid droplets were observed for glial knockdowns of the *Drosophila* orthologs of genes encoding enzymes converting fatty acids into lysophosphatidic acids (GPAT, CG3209, one of three glycerol-3-phosphate acyltransferases) or lysophosphatidic acids into phosphatidic acids (AGPAT, CG4753, one of four 1-acyl-sn-glycerol-3-phosphate acyltransferases) (Figure 4A; for enzyme predictions, see Wilfling et al., 2013). More strikingly, hypoxia-induced lipid droplets were reduced 95%–100% with knockdown of the enzymes converting phosphatidic acids into diacylglycerols (Lipin, 3-sn-phosphatidate phosphohydrolase), diacylglycerols into TAGs (DGAT1, a diacylglycerol O-acyltransferase), or with knockdown of Lsd-2, which promotes TAG storage in lipid droplets (Figures 4A and 4C). The RNAi results together suggest that TAGs are the predominant neutral lipids stored in glial droplets. They also demonstrate clearly that enzymes converting fatty acids into TAGs are required in glia for the hypoxic induction of lipid droplets. Importantly, there is a strong requirement for DGAT1, one of two enzymes catalyzing the final and only dedicated reaction in TAG synthesis, which is required for droplet biogenesis from the endoplasmic reticulum (Wilfling et al., 2014).

**Figure S4:**
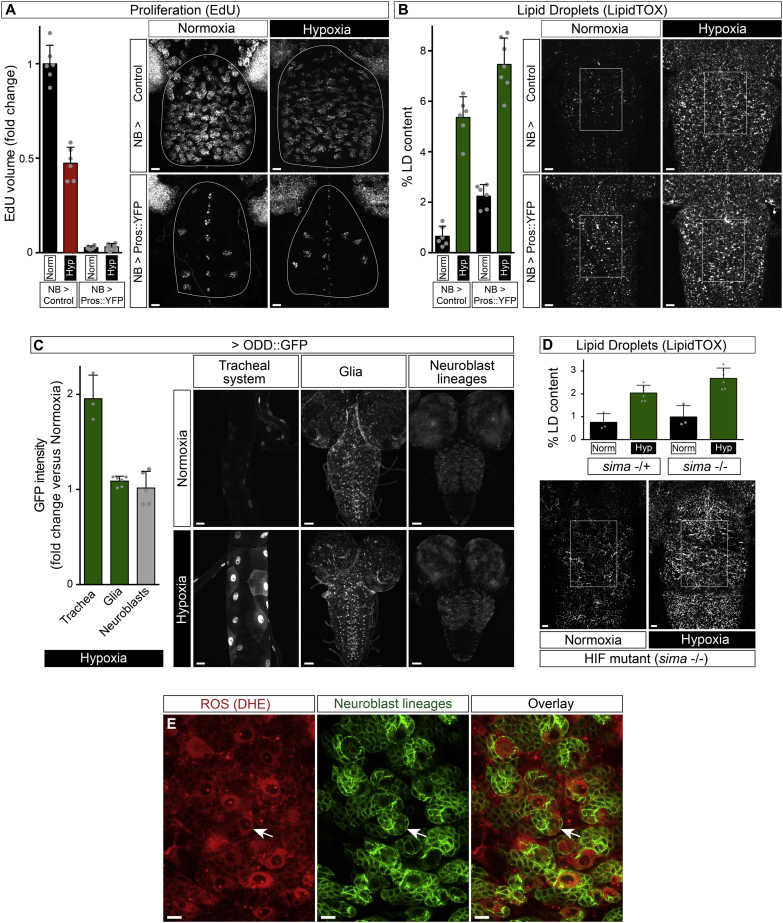
Hypoxic Induction of Lipid Droplets Does Not Require Neuroblast Proliferation or HIF Signaling, Related to Figure 5 (A) Pros::YFP misexpression in neuroblast lineages inhibits proliferation. In vivo EdU incorporation during normoxia or hypoxia is decreased in CNSs from NB > Pros::YFP (*elav*^*GMR71C07*^*, tubG80*^*ts*^*> Pros::YFP*) but not NB > control (*elav*^*GMR71C07*^*, tubG80*^*ts*^*>* ) larvae. Embryos were raised at 18°C and first instar larvae were then transferred to 29°C to alleviate Gal80 repression. Scale bars: 10 μm. (B) Blocking neuroblast proliferation does not inhibit the hypoxic induction of lipid droplets. Genotypes as in A. Scale bars: 10 μm. (C) Expression of a UAS-ODD::GFP reporter for stabilization of the oxygen-dependent degradation domain (ODD) of Hypoxia Inducible Factor 1 (HIF1). A 2.5 hr period of hypoxia (2.5% oxygen) significantly increases ODD::GFP intensity in the tracheal system (*btl > ODD::GFP*) but very little increase is observed in glia (*repo > ODD::GFP*) and no significant change is observed in neuroblast lineages (*elav*^*GMR71C07*^*> ODD::GFP*). Scale bars: Tracheal system, 20 μm; Glia and Neuroblast lineages, 50 μm. (D) Sima (HIF1) is not required for hypoxia induced CNS lipid droplets. Histogram shows comparable lipid droplet increases during hypoxia for heterozygous (*sima*^*07607*^*/TM6, Tb*) and homozygous (*sima*^*07607*^*/sima*^*07607*^) larvae. Scale bars: 10 μm. (E) Distribution of ROS in the CNS during normal development. ROS (oxidized DHE) were detected throughout the CNS, but at higher levels in neuroblasts (arrow), of a late third instar raised on standard diet in ambient normoxia. Neuroblast lineages were marked with GFP (*ase > CD8::GFP*). Scale bars: 10 μm.

To test whether the hypoxic induction of glial lipid droplets is functionally linked to neuroblast proliferation, we overexpressed YFP::Pros to drive dividing neuroblasts into premature differentiation (Maurange et al., 2008). This failed to inhibit glial lipid droplets, indicating that neuroblast proliferation is not required for glial lipid droplet induction during hypoxia (Figures S4A and S4B). The preceding RNAi results also allowed us to test for the reciprocal regulatory relationship, namely, that lipid droplets are required for neuroblast proliferation. Control experiments indicated no significant effects upon neuroblast proliferation if DGAT1 is knocked down in neuroblast lineages during normoxia or hypoxia, and also if DGAT1 is knocked down in glia during normoxia (Figures 4D and 4E; data not shown). However, glial-specific DGAT1 knockdown during hypoxia approximately halved neuroblast proliferation (Figure 4E). Together with the previous results, this finding demonstrates that the synthesis and storage of TAGs, as lipid droplets in niche glia, are required to sustain neuroblast divisions during hypoxia but not during normoxia.

### Reactive Oxygen Species Induce Glial Lipid Droplets

**Figure 5:**
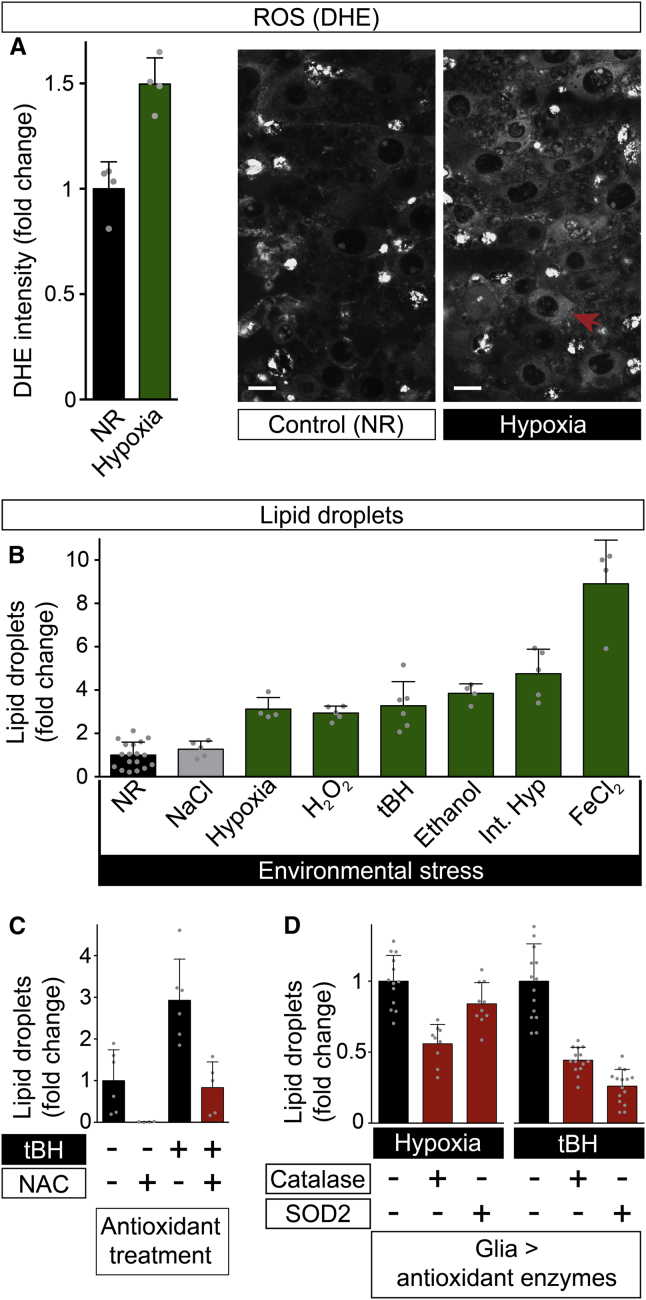
ROS Induce CNS Lipid Droplets (A) Hypoxia significantly increases ROS (measured by DHE oxidation) in many cells of the larval CNS including neuroblasts (red arrow marks one of the neuroblasts). Scale bars, 10 μm. (B) CNS lipid droplets increase (fold change relative to NR endpoint) in response to 22 hr of in vivo exposure to the following pro-oxidants: chronic hypoxia (2.5% oxygen), H_2_O_2_ (0.5% v/v), tBH (100 mM), ethanol (10% v/v), intermittent hypoxia (44 cycles of 10 min normoxia: 10 min anoxia) or FeCl_2_ (100 mM). Note that 22 hr of osmotic stress (1.25 M NaCl) does not significantly increase lipid droplets. (C) Dietary supplementation with the antioxidant N-acetyl cysteine (100 mM) inhibits lipid droplet induction by NR or tBH (100 mM). (D) Glial-specific overexpression of Catalase (*repo>Cat*) or Superoxide Dismutase 2 (*repo>SOD2*) inhibits lipid droplet induction by hypoxia (2.5% oxygen) or tBH (100 mM) (control genotype: *repo > w*^*1118*^). See also Figure S4.

To investigate the mechanism by which hypoxia induces glial lipid droplets, we first tested the HIF pathway. However, no substantial stabilization of a HIF::GFP fusion protein was observed in glia during hypoxia, and *sima* homozygotes lacking *Drosophila* HIF-1 activity retained the ability to accumulate lipid droplets during hypoxia (Figures S4C and S4D). We next considered alternative induction mechanisms. Hypoxia can markedly increase the levels of reactive oxygen species (ROS) generated by the mitochondrial electron transport chain, which, in turn, can lead to oxidative stress and cellular damage (Bleier and Dröse, 2013; Chandel et al., 1998; Olsen et al., 2013). Even in normoxic controls, the superoxide reactive dye dihydroethidium (DHE) detected more ROS in neuroblasts than in many other cells of the CNS (Figure S4E). Following hypoxia, ROS were moderately increased throughout the CNS, with higher levels detectable in neuroblasts (Figure 5A). We therefore tested whether intermittent hypoxia and a variety of chemical pro-oxidants known to increase ROS (hydrogen peroxide, tert-butyl hydroperoxide (tBH), ethanol, or iron (II) chloride) would be able to induce lipid droplets. All of these pro-oxidants induced glial lipid droplets at least as strongly as chronic hypoxia, whereas osmotic stress (1.25 M NaCl) did not (Figure 5B). The antioxidant N-acetyl cysteine (NAC) is a ROS scavenger and it replenishes the reduced glutathione pool (Atkuri et al., 2007). When added to the substrate, NAC inhibited both the moderate and strong increases of glial lipid droplets observed during NR and tBH stress, respectively (Figure 5C). In addition, glial-specific overexpression of the antioxidant enzymes catalase (Cat) or superoxide dismutase 2 (Sod2) inhibited hypoxic or tBH induction of glial lipid droplets (Figure 5D). Together, the chemical and genetic manipulations demonstrate that ROS are an important stimulus for inducing glial lipid droplets during normal development and in the presence of environmental pro-oxidants.

### PUFAs Redistribute from Phospholipids to TAGs during Oxidative Stress

**Figure 6:**
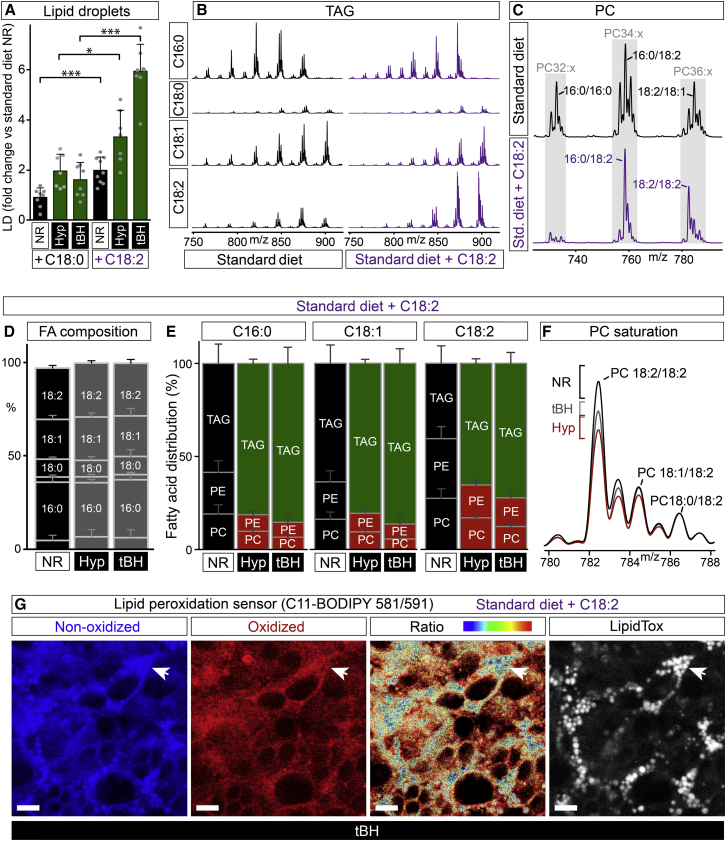
PUFAs Redistribute from Phospholipids to TAGs during Oxidative Stress (A) Lipid droplets following NR, hypoxia (2.5% oxygen), or tBH (100 mM) are more abundant on a diet supplemented with 20 mM linoleate (C18:2) than with 20 mM stearate (C18:0). (B) Neutral loss scans indicate that palmitate (C16:0), stearate (C18:0), oleate (C18:1), and linoleate (C18:2) are the major fatty acids present in CNS TAGs following NR. Dietary 20 mM linoleate (C18:2) leads to a relative enrichment of this PUFA in the TAG pool. X values are m/z, and Y values are relative signal intensities, scaled to the largest C16:0 peak. (C) Positive ion scans indicate the major PC mass envelopes (gray boxes) in the CNSs of NR larvae. Dietary 20 mM linoleate (C18:2) leads to a relative decrease in PC 16:0/16:0 and a relative enrichment of PC species containing C18:2. X values are m/z and Y values are relative signal intensities, scaled to the PC 16:0/18:2 peak. (D) Total FA composition (by GC-MS) of larvae raised on 20 mM linoleate diet. Neither hypoxia (n = 3) nor 100 mM tBH (n = 3) lead to major changes, compared to NR (n = 3), in the relative abundance of the major fatty acids (top to bottom: C18:2, C18:1, C18:0, C16:1, C16:0, and C14:0). (E) Relative distribution of major fatty acids (C16:0, C18:1, and C18:2) in the TAG, PC, and PE pools of CNSs from larvae on 20 mM linoleate diet. Hypoxia (n = 3) and 100 mM tBH (n = 4) both lead to a strong decrease, compared to NR (n = 3), in the proportion of all three major fatty acids in PCs and PEs, with a correspondingly strong increase in their proportions in TAG. (F) Positive ion scans of PC 18:x/18:x from the CNSs of larvae on a 20 mM linoleate diet. The ratio of 18:2/18:2 to 18:0/18:2 is decreased by hypoxia (n = 3, mean = 4.03, SD = 0.43, p = 0.008) and tBH (n = 3, m = 4.84, SD = 0.3, p = 0.09) relative to the NR control (n = 3, m = 5.7, SD = 0.62). For each treatment group, the mean spectrum is normalized to the 18:0/18:2 peak (m/z 786) of NR, and the maximum/minimum peak heights at m/z 782 (brackets) indicate variation between biological replicates. (G) CNS lipid peroxidation following 100 mM tBH treatment of larvae raised on a 20 mM linoleate diet. Co-incubation with the PUFA peroxidation sensor (C11-BODIPY 581/591) and LipidTOX 633 shows that the ratio of oxidized: non-oxidized sensor is lower in CNS lipid droplets (white arrow) than in cell membranes. Scale bars, 10 μm. See also Figure S5.

ROS attack the carbon-carbon double bonds in linoleic acid and other PUFAs, inducing lipid peroxidation chain reactions that can damage membrane phospholipids as well as other macromolecules (Negre-Salvayre et al., 2008). In the presence of oxidative stress (hypoxia or tBH), glial lipid droplets are induced more strongly when the diet is supplemented with linoleic (C18:2) than with stearic (C18:0) acid (Figure 6A). Mass spectrometry of isolated CNSs indicates that dietary supplementation with linoleic acid increases the proportion of this PUFA in the TAG, phosphatidylcholine (PC), and phosphatidylethanolamine (PE) pools (Figures 6B and 6C; data not shown). On the high-PUFA diet, neither hypoxia nor tBH detectably alter the total fatty acid profile of the CNS (Figure 6D). Both oxidative stresses do, however, produce a large decrease in the proportion of CNS linoleate in membrane phospholipids (PC and PE), with a correspondingly large increase in the proportion in TAG (Figure 6E). This redistribution from phospholipids to TAG is not selective for linoleate as it is also observed with the abundant monounsaturated and saturated fatty acids oleate (C18:1) and palmitate (C16:0) (Figure 6E). Together with our previous confocal microscopy and MIMS analyses, the mass spectrometry strongly suggests that oxidative stress leads to a bulk redistribution of fatty acids (including linoleic acid) from membranes to lipid droplets.

**Figure S5:**
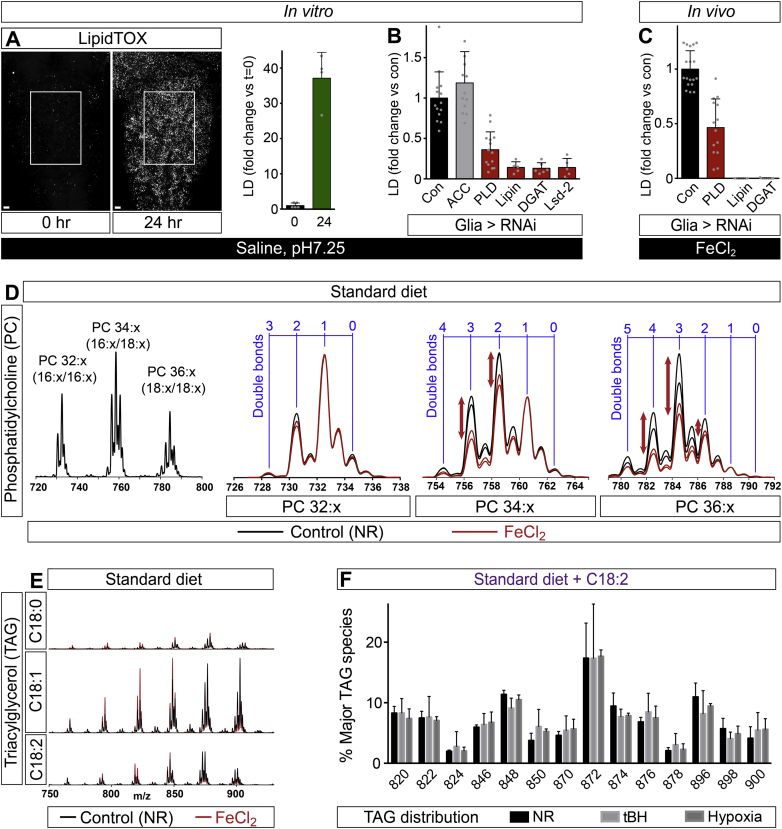
Iron Stress Selectively Depletes Linoleate from PCs, Related to Figure 6 (A) In vitro induction of neutral lipid puncta after 24 hr culture of the CNS in sugar-free saline. Note that the in vitro LipidTOX puncta are smaller than in vivo CNS lipid droplets. Scale bars: 10 μm. (B) In vitro induction of lipid droplets is inhibited by glial-specific knockdown of PLD, Lipin, DGAT1 or Lsd-2 (*repo>RNAi*, control: *repo > w*^*1118*^). (C) In vivo induction of CNS lipid droplets by 100 mM FeCl_2_ is inhibited by glial-specific knockdown of PLD, Lipin and DGAT1 (*repo>RNAi*, control: *repo > w*^*1118*^). (D) Tandem mass spectrometry of CNS phosphatidylcholines (PCs) from larvae subject to FeCl_2_ stress. Three major PC mass envelopes are shown at low (left) and high (right) resolution. High-resolution images compare the relative distributions of species containing different numbers of fatty acyl carbon-carbon double bonds under control (NR) and 100 mM FeCl_2_ stress. Two independent spectra for each condition are shown and the signal intensity (y axis) is normalized for each mass envelope to the species with 1 fatty acyl double bond. FeCl_2_ stress strongly decreases the relative abundance of PCs with at least one C18 chain (34:x and 36:x mass envelopes) and at least two C-C double bonds (red arrows). (E) Overlaid neutral loss scans from tandem mass spectrometry indicate that, in both control and FeCl_2_ conditions, CNS TAGs contain fatty acids that are saturated (18:0, stearic acid), monounsaturated (18:1, oleic acid) and polyunsaturated (18:2, linoleic acid). Y values correspond to relative signal intensities scaled to the largest C18:1 peak. (F) Tandem mass spectrometry shows that TAG composition does not change substantially between NR controls (n = 3), hypoxia (2.5% oxygen, n = 3) or tBH (100 mM, n = 4). Larvae were raised on the linoleate (C18:2) rich diet, and the relative abundance of the following TAG species was calculated: 16:1/16:1/16:0 (m/z 820), 16:1/16:0/16:0 (822), 16:0/16:0/16:0 (824), 18:2/16:1/16:0 (846), 18:2/16:0/16:0 (848), 18:3/18:2/16:0 and 18:2/18:2/16:1 (870), 18:2/18:2/16:0 (872), 18:1/18:2/16:0 (874), 18:1/18:1/16:0 and 18:2/18:0/16:0 (876), 18:1/18:0/16:0 (878), 18:2/18:2/18:2 (896), 18:2/18:2/18:1 (898), 18:2/18:1/18:1 and 18:2/18:2/18:0 (900). The *sn1* to *sn3* order of fatty acyl chains was not analyzed.

To identify how fatty acids are redistributed from CNS membranes to lipid droplets, we developed a simplified in vitro model that minimizes any input from fatty acid uptake or synthesis. Explanted CNSs were cultured in a saline medium lacking all lipids, sugars, and amino acids. This stress is sufficient to induce lipid droplets in an ACC-independent manner, but they are much smaller than those formed in vivo (Figures S5A and S5B). Nevertheless, droplet accumulation in vitro does require DGAT1 and Lsd-2, as it does in vivo (Figure S5B). Moreover, we found that the enzymes catalyzing the conversions of phosphatidylcholine (PC) into phosphatidic acid (PA) and PA into diacylglycerol (phospholipase D (PLD) and Lipin, respectively) are both required in glia for lipid droplet induction in vitro and in vivo (Figures 4A, S5B and S5C). Together, the in vitro and in vivo genetic analyses suggest that transfer of fatty acyl chains from membrane phospholipids to TAGs, via a PLD/Lipin/DGAT1 pathway, contributes to the biogenesis of glial lipid droplets during oxidative stress.

### Glial Lipid Droplets Protect Dividing Neuroblasts from PUFA Peroxidation

**Figure 7:**
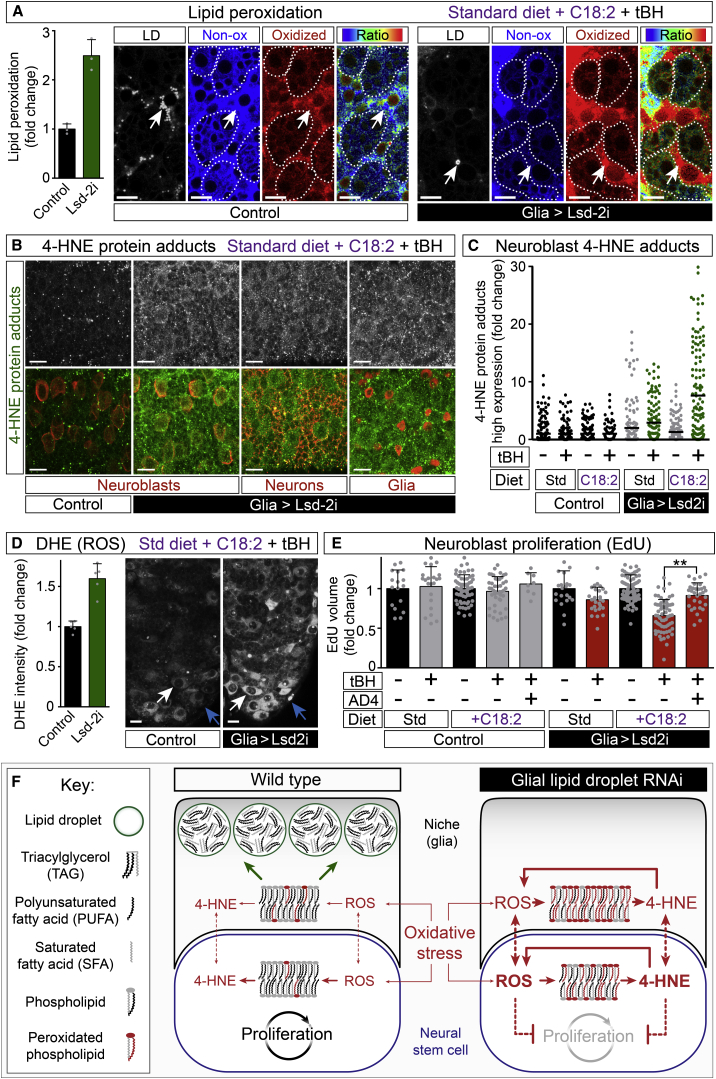
Lipid Droplets Protect Dividing Neuroblasts from PUFA Peroxidation (A) Lipid peroxidation following low-dose (10 mM) tBH treatment for 20 hr of larvae raised on a 20 mM linoleate (C18:2) diet. Co-incubation with the PUFA peroxidation sensor (C11-BODIPY 581/591) and LipidTOX 633 shows an increase in the ratio of oxidized: non-oxidized sensor in neuroblast lineages (dotted outlines) and also in glia following the knockdown of lipid droplets (*repo > Lsd-2 RNAi*, control genotype: *repo > w*^*1118*^). With *repo > w*^*1118*^, there is a low peroxidation ratio in LipidTOX^+^ droplets (arrow), but, with *repo > Lsd-2 RNAi*, there is a high peroxidation ratio in rare abnormal LipidTOX^+^ puncta (arrow). The same two genotypes are used in (B)–(E). Scale bars in this and subsequent panels, 10 μm. (B) Glial lipid droplet knockdown increases 4-HNE protein adducts in many cells of the CNS, including neuroblasts (marked with anti-Miranda), neurons (marked with anti-Neurotactin), and glia (marked with anti-Repo). (C) Quantification of 4-HNE protein adducts in neuroblasts. Glial lipid droplet knockdown in the presence of 10 mM tBH (+ tBH), but not in NR controls (– tBH), leads to a significant increase in 4-HNE protein adducts. This is further increased when larvae are raised on a 20 mM linoleate diet. Mann-Whitney test: gray p > 0.1, green p < 0.001 relative to the appropriate genotype control. (D) ROS increase following glial lipid droplet knockdown. Oxidized DHE levels increase significantly throughout the CNS (histogram) with elevated levels in glia (blue arrows) and in neuroblasts (white arrows). (E) Neuroblast proliferation is significantly inhibited by 10 mM tBH if glial lipid droplets are knocked down. Inhibition is stronger when larvae are raised on a 20 mM linoleate diet and can be significantly rescued by dietary supplementation with 40 μg/ml AD4. In the control genotype, tBH and 20 mM dietary linoleate do not significantly decrease neuroblast proliferation. Pooled data are represented as mean ± SD. (F) PUFA protection model for the antioxidant role of lipid droplets. The neural stem cell niche (glia) and the neural stem cell (neuroblast) are depicted in the presence of oxidative stress, in a CNS that is wild-type (left) or RNAi knockdown for a glial lipid droplet gene (right). Oxidative stress stimulates the biogenesis of glial lipid droplets that protect vulnerable PUFAs in the TAG core from ROS-induced peroxidation. Peroxidation of omega-6 PUFAs produces 4-HNE, which, in turn, can generate more ROS, leading to PUFA chain reactions that damage membrane lipids and other macromolecules such as proteins. Following oxidative stress, the proportion of total PUFAs and other fatty acids increases in the TAG pool, relative to the phosphatidylcholine pool. This may correspond to bulk redistribution of lipids from membranes to lipid droplets, which would decrease the amount of membranes and thus minimize the fraction of PUFAs that are susceptible to ROS-induced peroxidation. The mechanism by which ROS and/or 4-HNE inhibit stem cell proliferation and the molecule(s) communicating between the niche and the stem cell are not yet clear (dotted arrows). See Discussion for details. See also Figure S6.

On the high PUFA diet, oxidative stress from tBH or hypoxia selectively depletes linoleic acid (relative to monounsaturated and saturated fatty acids) from the PC pool of the CNS (Figure 6F). On a standard diet in the presence of iron (II), a strong pro-oxidant (Olsen et al., 2013; Figure 5B), selective depletion of linoleate from PCs was also observed (Figure S5D). Although linoleic acid is selectively lost from the PC pool during oxidative stress, we failed to detect its selective increase (or decrease) in the TAG fraction during iron, tBH, or hypoxic stress (Figures S5E and S5F). Hence, the loss of linoleate from PCs may not reflect its selective remobilization to lipid droplets but could result from its selective susceptibility (over more saturated fatty acids) to ROS-induced peroxidative damage. Indeed, tBH stress on a high linoleate diet does lead to increased PUFA peroxidation, as detected by a ratiometric fluorescent sensor (C11-BODIPY 581/591, Figure 6G). Strikingly, the ratio of oxidized to non-oxidized sensor in the CNS is much higher in cell membranes than in lipid droplets. Importantly, the PUFA peroxidation ratio of CNS membranes increases even further if lipid droplet accumulation is abrogated by Lsd-2 knockdown (Figure 7A). Even though the knockdown was glial specific and a very strong increase in PUFA peroxidation was observed in glial membranes, a moderate increase in PUFA peroxidation was also detected in the membranes of neuroblast lineages. This indicates that glial lipid droplets exert a non-cell autonomous and protective effect upon neuroblast lineages. Together with the mass spectrometry, the fluorescent sensor provides evidence that PUFAs like linoleic acid are more susceptible to peroxidation when incorporated into membrane phospholipids than when stored as TAGs in the core of lipid droplets.

**Figure S6:**
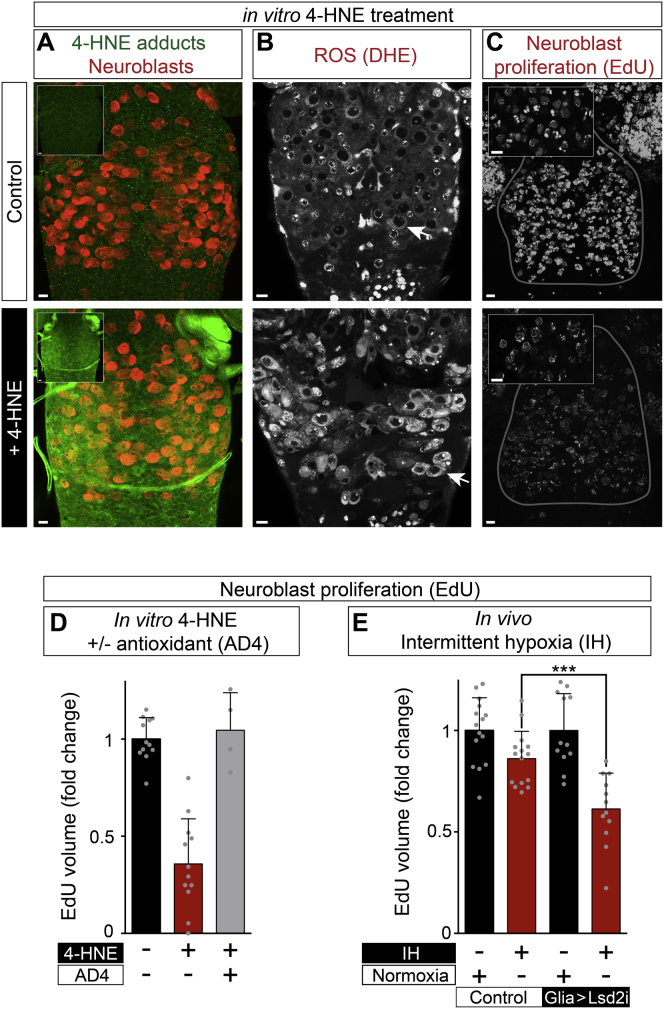
Exogenous 4-HNE Increases ROS and Inhibits Neuroblast Proliferation, Related to Figure 7 (A) In vitro incubation with 4-HNE (100 μM for 1 hr) generates 4-HNE protein adducts in cells throughout the CNS, including neuroblasts (marked with Miranda, red). Scale bars: 10 μm. (B) In vitro incubation with 4-HNE (100 μM for 30 min) increases ROS (DHE oxidation) in the CNS, particularly in neuroblasts (arrows). Scale bars: 10 μm. (C) In vitro incubation with 4-HNE (100 μM for 2 hr) decreases neuroblast proliferation (EdU incorporation into neuroblast lineages). Main panels show confocal projections, insets show single confocal sections at a higher magnification. Scale bars: 10 μm. (D) Quantification of 4-HNE inhibition of neuroblast proliferation and rescue by the antioxidant AD4 (40 μg/ml). Conditions as in C. (E) In vivo neuroblast proliferation is inhibited by intermittent hypoxia, weakly in a control genotype (*repo > w*^*1118*^*)* and more strongly when glial lipid droplets are knocked down (*repo > Lsd-2 RNAi*). Intermittent hypoxia corresponds to 44 cycles of 5 min anoxia, 25 min normoxia.

PUFA peroxidation can also lead to the damage of non-lipid macromolecules. For example, peroxidation chain reactions of omega-6 PUFAs such as linoleic acid generate 4-hydroxy-2-nonenal (4-HNE), an unsaturated aldehyde that forms covalent adducts with proteins (Uchida, 2003). Following in vitro incubations with exogeneous 4-HNE, such protein adducts can be detected by immunocytochemistry in most/all cells of the CNS, including neuroblasts (Figure S6A). Consistent with studies in other biological contexts (Uchida, 2003), 4-HNE induced ROS throughout the CNS but, as seen previously during hypoxia and even during normal development, levels were higher in cells of a large size characteristic of neuroblasts (Figure S6B). 4-HNE also markedly decreased the proliferation of neuroblasts, but this was efficiently rescued with the antioxidant N-acetylcysteine amide (AD4) (Figures S6C and S6D). These in vitro CNS experiments reveal that 4-HNE and ROS induce each other and also that high levels of one or both are deleterious for neuroblast proliferation.

We next determined in vivo whether glial lipid droplets function not only to defend membrane lipids against peroxidation, but also to protect proteins from 4-HNE damage. For the control genotype, no obvious increase in 4-HNE protein adducts was observed with tBH stress on standard or high PUFA diets (Figures 7B and 7C). In contrast, for glial Lsd-2 knockdown, tBH stress increased 4-HNE protein adducts significantly in neuroblasts, and this increase was further augmented by a high PUFA diet (Figures 7B and 7C). Cell-type markers indicated that Lsd-2 knockdown increases 4-HNE protein adducts not only in neuroblasts, but also in glia and in neurons (Figure 7B). Under these in vivo conditions, glial lipid droplet knockdown led to increased ROS throughout the CNS, with maximal levels in neuroblasts (Figure 7D). These findings demonstrate that glial lipid droplets are required to defend the proteins of neuroblasts and other neural cells against 4-HNE damage from even a moderate oxidative challenge.

Finally, we tested whether a high PUFA diet influences the ability of glial lipid droplets to protect neuroblast proliferation. With intermittent hypoxia on a high PUFA diet, a slight decrease in neuroblast proliferation was observed, and this was exacerbated with glial Lsd-2 knockdown (Figure S6E). The milder oxidative stress of tBH on a high PUFA diet (or on a standard diet) did not significantly alter proliferation nor detectably increase CNS apoptosis in larvae of the control genotype (Figure 7E; data not shown). In larvae with glial knockdown of Lsd-2, however, proliferation was decreased slightly by tBH and much more strongly by the combination of tBH with high dietary PUFA (Figure 7E). This deficit in proliferation could be significantly rescued by dietary supplementation with the antioxidant AD4. Hence, when the diet is rich in PUFAs, glial lipid droplets play a particularly critical role in sustaining the proliferation of neuroblasts during oxidative stress.

## Discussion

This study reveals that the proliferation of *Drosophila* neural stem cells is defended against oxidative stress and identifies lipid droplets in niche glia as a critical element of the protective mechanism. During oxidative stress, PUFAs and other fatty acids are redistributed from membrane phospholipids to lipid droplet TAGs. Unlike cell membranes, the lipid droplet core provides a protective environment that minimizes PUFA peroxidation chain reactions and limits ROS levels. This helps to safeguard not only the glial cells of the niche but also the neighboring neural stem cells and their neuronal progeny. We now compare the protective roles of the niche glia during oxidative stress and starvation, discuss the lipid droplet antioxidant mechanism, and speculate upon its wider implications.

### Niche Glia Protect Neuroblasts from Oxidative Stress and Nutrient Deprivation

For both NR and oxidative stress, the protection of neuroblast divisions involves niche glia. We previously showed that neural growth and proliferation are spared during NR by constitutive glial secretion of Jeb, a ligand for Alk/PI3-kinase signaling in neuroblasts (Cheng et al., 2011). In the present study, we find that neuroblast proliferation is also spared during oxidative stress and that this requires the glial activity of proteins involved in TAG metabolism such as DGAT1 and Lsd-2. These enzymes promote the accumulation of PUFA and other fatty acids as lipid droplet TAGs in cortex and subperineurial glia. TAG synthesis is required in these niche glia for sustaining neuroblast proliferation during oxidative stress but this is not the case during NR or fed conditions. Hence, although niche glia are critical for protecting neighboring neuroblasts from nutrient deprivation and from oxidative stress, in each case a different molecular mechanism is involved. Presumably both glial mechanisms work together to give neuroblasts the remarkable ability to continue dividing even when oxidative stress is combined simultaneously with NR.

### A High PUFA Diet Increases the Vulnerability of Neuroblasts to ROS Damage

We found that a high PUFA diet makes neural stem cells and their glial niche more vulnerable to ROS-induced damage. Omega-3 and omega-6 PUFAs are obtained from the diet as both *Drosophila* and humans lack the key desaturases required to synthesize them. These PUFAs are essential fatty acids for humans but they do not appear to be required for *Drosophila* survival (Rapport et al., 1983). Larvae do, however, consume significant quantities of the omega-6 PUFA linoleic acid and increasing its concentration in the diet makes neuroblasts and other developing neural cells more susceptible to damage from ROS-induced lipid peroxidation chain reactions. Glial lipid droplets make an important contribution toward minimizing this ROS damage in the CNS by storing linoleic acid and protecting it from peroxidation. Saturated and monounsaturated fatty acids are much less vulnerable than PUFAs to peroxidation, but they too accumulate in glial lipid droplets during oxidative stress. Whether these fatty acids contribute to the protection of PUFAs in lipid droplets is not yet clear but, in the membranes of cancer cells, they are thought to decrease ROS damage by diluting PUFAs in the phospholipid pool (Rysman et al., 2010). It may also be important to avoid excess saturated fatty acids in membrane phospholipids, and the scavenging of extracellular monounsaturated lysophospholipids provides one way of preventing this (Kamphorst et al., 2013).

### An Antioxidant Role for the Lipid Droplet

It is becoming increasingly clear that lipid droplets mediate cellular functions other than fat storage and mobilization relevant for energy homeostasis. For example, lipid droplets can participate in protein degradation, histone storage, viral replication, and antibacterial defense (Anand et al., 2012; Walther and Farese, 2012). Our study now reveals an additional role for lipid droplets as an antioxidant organelle, defending membrane PUFAs from damage by ROS-induced peroxidation. We also found that lipid droplets play a wider antioxidant role, protecting cellular proteins from reactive peroxidation products such as 4-HNE. The results presented here support a PUFA protection model for the antioxidant mechanism of lipid droplets (Figure 7F). Central to this model are five key findings. First, DGAT1 and Lsd-2 knockdowns and AD4 rescues show that glial lipid droplets act during oxidative stress to minimize ROS, PUFA peroxidation, and 4-HNE damage and also to sustain neuroblast proliferation. Second, lipid peroxidation sensor and tandem mass spectrometry experiments argue that the major diet-derived PUFA, linoleic acid, is less vulnerable to peroxidation in lipid droplet TAGs than in membrane phospholipids. Third, Catalase or SOD2 overexpression or NAC supplementation provides evidence that ROS (directly or indirectly) induce lipid droplets during oxidative stress. Fourth, MIMS, tandem mass spectrometry, PLD knockdown, and CNS explants demonstrate that saturated, monounsaturated, and polyunsaturated fatty acids redistribute from membrane phospholipids to lipid droplet TAGs during oxidative stress. And fifth, in vitro and in vivo experiments together show that pro-oxidants not only stimulate widespread ROS and PUFA peroxidation but also that 4-HNE, a product of PUFA peroxidation, is itself capable of increasing ROS, particularly in neuroblasts. This identifies a positive feedback loop in the CNS between ROS, PUFA peroxidation, and 4-HNE but also suggests that it will be difficult to pinpoint the molecule(s) propagating oxidative damage between glia and neuroblasts.

Our findings raise the important issue of why PUFAs are more efficiently protected from peroxidation in the core of lipid droplets than in membranes. Future biophysical investigations will be needed to resolve this but it is likely that lipid droplets provide a means to segregate reactive PUFAs away from aqueous pro-oxidants and lipid peroxidation chain reactions at membrane-aqueous interfaces. Studies of oil-and-water emulsions indicate that TAGs in small lipid droplets are less mobile and thus better protected from peroxidation chain reactions than those in larger lipid droplets (Nakaya et al., 2005). Interestingly, lipid droplets in glia are smaller, on average, than those found in the major lipid storage depot of the fat body. Given this, and that their biogenesis in glia requires DGAT1, it is interesting that a previous study found that DGAT1 and DGAT2 are involved in the formation of small versus large lipid droplets, respectively (Wilfling et al., 2013).

Lipid droplets have been observed in many different cell types challenged with hypoxia, ischemia, or various metabolic imbalances. For example, mitochondrial protein knockdowns in *Drosophila* photoreceptor neurons generate ROS and induce lipid droplets in neighboring pigment and epithelial glia (Liu et al., 2015). In this mutant context, either neuronal or glial-specific overexpression of lipases can decrease lipid droplets and ameliorate photoreceptor degeneration. In a mammalian example, hypoxia induces lipid droplets in glioblastoma and breast cancer cells via a pathway involving HIF-1α (Bensaad et al., 2014). This hypoxic induction mechanism in cancer cells appears different from the HIF-1 independent pathway that we have identified in the *Drosophila* neural stem cell niche. It is nevertheless intriguing that, in both contexts, inhibiting lipid droplet formation increases ROS toxicity and impairs cell proliferation. It will therefore be important in future to determine whether lipid droplets also play antioxidant roles in other biological contexts.

## Experimental Procedures

### Larval Environmental Manipulations and EdU Labeling

Larvae at 0–6 hr after larval hatching (ALH) were raised, 20 per vial, at 25°C on standard cornmeal/yeast/agar food (composition in Gutierrez et al., 2007) until mid-third instar (66–72 hr ALH), then subjected to environmental stresses. For NR, larvae were floated from standard diet using 30% glycerol/PBS and then transferred to 0.5% low melting-point agarose. N-acetyl cysteine (pH 7.2) and/or pro-oxidants were added to agarose at 38°C, before cooling. Stearic or linoleic acid (Sigma-Aldrich) was supplemented to 20 mM in standard food. Hypoxia used an O_2_ Cabinet (Coy Lab Products) calibrated to 0% and 20.9% oxygen, with 2.5% oxygen maintained by automated nitrogen injection. Intermittent hypoxia used alternate injections of nitrogen and air over multiple cycles. For the in vitro EdU assay, dissected tissues were incubated at 25°C for 1 hr in PBS with 10 μm EdU. For the in vivo EdU assay, larvae were transferred to 0.5% low melting-point agarose with 100 μm EdU for 3 hr and stresses then applied in the presence of EdU for a further 19 hr.

### Tissue Imaging and Analysis

Tissues were fixed and stained for EdU with the Click-iT EdU Alexa Fluor 555 Imaging Kit (Molecular Probes) and for neutral lipids using oil red O or LipidTOX (Molecular Probes). Live ex vivo imaging was used to detect oxidized DHE (Invitrogen) and peroxidated lipid (C11-BODIPY 581/591, Invitrogen). See Supplemental Experimental Procedures for details.

### Lipid Analysis by MIMS, Tandem MS, and GC-MS

For Multi-Isotope Imaging Mass Spectrometry (MIMS), larvae were fed diets containing 1-^13^C Glucose, 1-^13^C acetate, or ^13^C-U-linoleic acid (Cambridge Isotope Laboratories), and after hypoxia (2.5% oxygen for 22 hr) CNSs were dissected, fixed, embedded in Technovit 7100 (Heraeus Kulzer), and sectioned at 2 μm. Analysis was performed on 30- to 50-μm regions of interest with simultaneous imaging of multiple ions, including ^12^C^14^N, ^12^C, and ^13^C. Tandem mass spectrometry (nanospray infusion) and gas chromatography mass spectrometry were performed on lipid extracts of 6-15 CNSs. See Supplemental Experimental Procedures for details.

## Author Contributions

A.P.B. and A.P.G. designed the experiments and wrote the paper. A.P.B. conducted *Drosophila* experiments, MIMS sections were prepared by E.M.A.H. and analyzed by C.G. and C.P.L., and lipid extracts were analyzed by tandem mass spectrometry (G.K. and A.D.P.) and GC-MS (J.I.M.).

## Supplemental Information

Document S1. Supplemental Experimental Procedures

Movie S1. Drosophila Larvae Exit the Food during Hypoxia, Related to Figure 1 Time-lapse images of larval behavior during intermittent hypoxia were taken at 10 s intervals, and the time-stamp is shown in min. Hypoxia was progressively induced under a stream of nitrogen and larvae were rapidly returned to normoxia by removing the lid of the chamber. The timings of the cycles were hypoxia (0–6 min), normoxia (6–16 min), hypoxia (16–27 min), and then normoxia (27–42 min).

Document S2. Article plus Supplemental Information
