# Supplementary material for: Antioxidant Role for Lipid Droplets in a Stem Cell Niche of Drosophila
Source: Cell. 2015 Oct 8;163(2):340–53. doi: 10.1016/j.cell.2015.09.020 (PMC4601084; doi:10.1016/j.cell.2015.09.020)
Supplement: Document S1. Supplemental Experimental Procedures [file mmc1.pdf]

Cell

Supplemental Information

Antioxidant Role for Lipid Droplets in a Stem Cell Niche of *Drosophila*

Andrew P. Bailey, Grielof Koster, Christelle Guillermier, Elizabeth M.A. Hirst, James I. MacRae, Claude P. Lechene, Anthony D. Postle and Alex P. Gould

SUPPLEMENTAL EXPERIMENTAL PROCEDURES

Drosophila genetics

Genetic elements are described in Flybase (St Pierre et al., 2014) (http://flybase.org/). The control genotype used for environmental and GAL4/UAS manipulations was $w^{1118}{}_{iso}$; 2_{iso}; 3_{iso} (iso 31) (Ryder et al., 2004) unless otherwise stated. Genetic elements used were *repo-Gal4* (Xiong et al., 1994), *nab-Gal4*^{NP1316} (Maurange et al., 2008), *elav-Gal4*^{C155}, *elav-Gal4*^{GMR71C07}, *nrt-Gal4*^{GMR37H03}, *ase-Gal4* (Zhu et al., 2006), *Moody-Gal4* (Schwabe et al., 2005), *tubP-GAL80*$^{ts[7]}$ (McGuire et al., 2003), *UAS-CD8::GFP*, *UAS-Lsd-2::GFP* (kindly provided by Michael Welte), *Lsd-2::YFP*^{CPTI001655} (DGRC), *UAS-Pros::YFP* (Choksi et al., 2006), *UAS-ODD::GFP* (Arquier et al., 2006), *sima*^{07607} (Centanin et al., 2005). UAS-RNAi lines were obtained from VDRC (TAG synthesis genes described in Kuhnlein, 2012): *CG11198*^{KK108631} (ACC), *CG3524*^{KK105855} (FAS ortholog), *CG3209*^{KK100728} (GPAT ortholog), *CG4753*^{KK109865} (AGPAT ortholog), *CG8709*^{KK107707} (Lipin), *CG31991*^{KK100003} (DGAT1), *CG9057*^{KK102269} (Lsd-2) and *CG12110*^{KK106137} (PLD).

EdU detection

Following EdU administration, tissues were fixed overnight at room temperature in 2% paraformaldehyde in PBL (75 mM lysine, 37 mM sodium phosphate buffer at pH7.4), rinsed in PBS, and adhered to poly-L lysine coated slides for subsequent steps. After permeabilization in PBST (PBS with 0.2% Triton X-100) tissues were blocked in 10% normal goat serum in PBST, and EdU was detected using the Click-iT EdU Alexa Fluor 555 Imaging Kit (Life Technologies). Following further washes in PBST and PBS, tissues were mounted in Vectashield (Vector Laboratories), and imaged using a Leica SP5 confocal microscope.

Lipid droplet staining

To image neutral lipids, tissues were fixed overnight in 2% paraformaldehyde in PBL, then washed in PBS. Oil Red O staining was performed as previously described (Gutierrez et al., 2007), and images were acquired using a Zeiss Axiophot 2 compound microscope. To image the overall lipid droplet content of the CNS or wing disc, fixed

tissues were adhered to poly-L lysine coated slides, permeabilized for 5 min in PBST, washed extensively in PBS (with 1 mg/ml DAPI where required), and stained overnight at room temperature in LipidTOX Green (HCS LipidTOX Neutral Lipid Stain, Life Technologies) at 1:1000 dilution. Following one rinse in PBS, tissues were rinsed and mounted in Vectashield for confocal microscopy. To visualize lipid droplet morphology in conjunction with the Lsd-2::YFP lipid droplet marker, the CNS was fixed as above, stained overnight with DAPI and LipidTOX Red (1:1000), and mounted in PBS to preserve lipid droplet morphology. To prepare cross sections of GFP expressing tissues, samples were fixed overnight in 2% paraformaldehyde in PBL, embedded in BSA/gelatin/formaldehyde (Levin, 2004) and sectioned at 40 μm using a Leica VT 1000S vibratome. DAPI and LipidTOX Red staining was then performed as described above, and sections were mounted in PBS for confocal imaging.

Analysis of EdU and lipid droplet volumes

For volume measurements of EdU or LipidTOX in the CNS, a Leica SP5 was used to acquire image stacks (32 x 1 μm, 40x objective) of the cortical region of the ventral nerve cord. No offset was used, and the gain was adjusted for each sample to standardize intensity distribution across the dynamic range of the PMT detector (0-255). For lipid droplet measurements, stacks were cropped to a fixed cuboidal region of interest (ROI) covering the center of the thoracic ganglion. Total lipid droplet volume was quantified in Volocity (PerkinElmer), measuring only those objects above a minimal size (1 μm^3) and intensity. A similar method was used for EdU quantification, except that the ROI was defined for each sample, encompassing the proliferating cells of the thoracic ganglion of the ventral nerve cord, and the minimum object size was set at 3 μm^3. For wing discs, equivalent measurements were taken from the whole organ volume. All quantifications were performed on raw images but, for presentational clarity, adjustments of brightness and contrast were applied equally to all panels of a given figure.

Immunocytochemistry

Tissues were fixed for 40 min at room temperature in 2% paraformaldehyde in PBL, then washed in PBS and adhered to poly-L-lysine slides. After permeabilization in PBST,

tissues were blocked for 1 hr in PBST containing 10% NGS, then primary antibodies were added in block solution and incubated overnight at 4ºC. After extensive washes in PBST, block solution containing secondary antibodies and 1 mg/ml DAPI was added for 2hr at room temperature, then washed extensively in PBST and mounted in Vectashield (Vectorlabs) for imaging. Primary antibodies were used at the following dilutions: 4-HNE protein adducts (1:100, Abcam 46545), Miranda (1:50, gift of F. Matsuzaki), Repo (1:20, DSHB 8D12), Neurotactin (1:20, DSHB BP106). Secondary antibodies: Alexa Fluor labelled Fab fragments (1:1000, Life Technologies).

Quantification of oxidized DHE, 4-HNE-adducts and lipid peroxidation

Dihydroethidium (DHE, Life Technologies) staining was performed as described (Owusu-Ansah et al., 2008). Briefly, tissues were dissected in Schneider's medium, incubated for 30 min in 30 μM DHE, then washed and mounted in Schneider's medium for confocal imaging. Pairs of control and experimental CNS samples were imaged simultaneously by confocal microscopy using a 10x objective, and staining intensity was quantified in the thoracic ventral nerve cord using Volocity (PerkinElmer). Images for figure panels were captured under the same settings using the 40x objective. For 4-HNE adduct staining (using Alexa 488 secondary antibodies, Life Technologies) control and experimental CNS samples were processed together, and a single confocal section was acquired for each ventral nerve cord using a 40x objective (5 CNSs per treatment group). Neuroblasts were identified by morphological criteria and marked in Volocity with a circular ROI of fixed size from which the fluorescence intensity per neuroblast was measured at 500-550 nm emission. For each neuroblast, the number of pixels with intensity between 175-255 was quantified and data points show the fold change relative to the mean control value. To detect lipid peroxidation in lipid droplets and in cell membranes, CNSs were dissected in Schneider's medium and incubated for 30 min in Schneider's medium containing 10% FBS, LipidTox Deep Red (1:200) and 2 μM C11-BODIPY 581/591 (Invitrogen, D3861). Samples were washed and mounted in Schneider's medium and then control/experimental samples were imaged alternately to detect the non-oxidized (excitation: 561 nm, emission: 570-610 nm) and oxidized (excitation: 488 nm, emission: 500-540 nm) forms. The oxidized: non-oxidized ratio was

measured in each CNS from a 90 µm x 90 µm ROI in the thoracic ganglion, and intensity modulated ratiometric images were generated using Volocity software.

CNS explant cultures and 4-HNE treatment

Mid third instar larvae were dissected in saline (containing penicillin and streptomycin, pH 7.25, Yoshihara et al., 2000), ensuring that the eye-antennal discs, leg discs and ring gland were removed without CNS damage. Each CNS was cultured individually in 1 ml saline at 25 ºC for 22 hr, then fixed and stained with LipidTOX neutral lipid stain (Life Technologies). For *in vitro* 4-HNE treatment, mid third instar larvae were dissected in Schneider's medium and incubated in Schneider's medium containing penicillin and streptomycin, 10% FBS and 100 µM 4-HNE (Biovision, #2083). After 1 hour, samples were fixed and processed for immunocytochemical detection of 4-HNE adducts. To measure the effects of 4-HNE on ROS production, CNSs were incubated for 30 min in 100 µM 4-HNE with 30 µM DHE, then washed and mounted in Schneider's medium. Control and experimental samples were imaged alternately by confocal microscopy. 4-HNE inhibition of neuroblast proliferation was assayed by adding 10 µM EdU to the Schneider's medium, in the presence or absence of 40 µg/ml AD4 (Sigma Aldrich). After 2 hr, samples were fixed and processed for EdU incorporation as above. 4-HNE was supplied as a 10 mg/ml solution in ethanol so, in all cases, the equivalent dilution of ethanol (1.55 µl/ml) was used as a control.

Multi-Isotope Imaging Mass Spectrometry

For dietary glucose incorporation, the glucose component of the standard diet was replaced with 1-^{13}C glucose (Sigma Aldrich), to a final concentration of 325 mM. To assess dietary acetate incorporation, 1-^{13}C acetate (Sigma Aldrich) was added to standard unlabeled food at a final concentration of 170 mM. To measure linoleate incorporation, the standard diet was supplemented with 20 mM of a 1:1 mix of ^{13}C-U-linoleic acid (Cambridge Isotope Laboratories) and unlabeled linoleic acid. Larvae were raised on labeled food until mid third instar, then transferred to NR medium in hypoxia (2.5% oxygen) for 22 hr. Partially dissected larvae were then fixed overnight in 2% paraformaldehyde in PBL, washed in PBS then transferred to sodium cocodylate buffer

(SCB, 0.1 M, pH7.2). After post-fixing overnight in SCB/ 2% formaldehyde/ 2% glutaraldehyde, larvae were washed in SCB and transferred to 2% osmium tetroxide in SCB for 2 hr. After further washes with SCB, the CNS was dissected and embedded in 1.5% low melting point agarose to aid orientation during sectioning. The block was dehydrated through an ethanol series, incubated for 1 hr in 1:1 ethanol:Technovit 7100 solution A, then incubated overnight in Technovit 7100 solution A. The Technovit block was polymerized according to the manufacturer (Heraeus Kulzer), and 2 μm sections were cut on a Ultracut E microtome (Reichert Jung). Sections were mounted on silicon chips and analyzed for MIMS as previously described (Steinhauser et al., 2012, see online methods: http://dx.doi.org/10.1038/nature10734).

Lipidomics

A previously described protocol (MacRae et al., 2013) was adapted for *Drosophila* CNS samples. Briefly, CNSs were dissected from NR, hypoxic or tBH treated larvae in ice-cold ammonium acetate (150 mM) taking care to remove associated tissues (eye-antennal discs, leg imaginal discs and ring gland). Groups of 6 CNSs were homogenized by squeezing the tissues between two small glass coverslips. The homogenized samples on glass were transferred to 400 μl chloroform, then 200 μl methanol was added (in the presence of 20 mg/ml butylated hydroxytoluene), followed by three bursts of bath sonication for 10 min each at 4ºC. For $FeCl_2$ stress, groups of 15 CNSs were transferred directly to chloroform:methanol (2:1 v/v) for extraction. Samples were then dried under N_2, re-extracted using 600 μl methanol:water (2:1 v/v), bath sonicated (3x10 min), added to the chloroform:methanol extract, and then dried under N_2. Biphasic partitioning was achieved by the addition of 700 μl chloroform:methanol:water (1:3:3 v/v) and, after centrifugation (5 min), the lower phase was removed and dried under N_2.

For tandem mass spectrometry, lipid extraction was performed in the presence of the following lipid standards: TAG (C14:0/C14:0/C14:0), PC (C14:0/C14:0), PE (C14:0/C14:0). Following biphasic partitioning, lipid extracts were dissolved in 25 μl of methanol: dichloromethane: water: concentrated ammonia (66:30:3:1, v:v) and infused by syringe pump at 80 nl/min directly into the nanospray interface of a XEVO TQ mass

spectrometer (Waters, Milford Massachusetts). A portfolio of spectra were collected for all samples, including precursor scans of m/z 184 (diagnostic for PC) and m/z 141 (diagnostic for PE). PC and PE compositions were determined from negative ion scans and fatty acids in TAG molecular species were determined from neutral loss scans of fatty acyl fragments under positive ionization. Following correction for natural isotopic abundance, peak heights were quantified with reference to the appropriate external standard. To quantify fatty acid distribution among the TAG, PC and PE fractions, the abundance of a given fatty acid within each species was weighted according to its composition (for example, C18:2 is twice as abundant in PC 18:2/18:2 as in PC 16:0/18:2). The total amount of each FA in the combined TAG/PC/PE pools was then calculated for each sample and set to 100%, from which the relative distribution within each fraction could be calculated. Saturation changes within the PC 18:x/18:x envelope were calculated from the peak height ratio of PC 18:2/18:2 to PC 18:2/18:0.

For GC-MS measurements of total fatty acid composition and ^{13}C labeling, lipid extractions were performed as above but no standards were added during extraction. Biphasic partitioning was performed in the presence of an external standard (25 nmol $^{13}C_1$-dodecanoic acid, C12:0), and the apolar faction dried and subsequently washed twice with methanol. Dried residues were then dissolved in 25 μL chloroform/methanol (2:1), 5 μL Methprep II (Grace Davison, Alltech) added, and fatty acid methyl esters (FAMEs) were analyzed directly on an Agilent 7890B-5977A GC-MS system. Data analysis and peak quantifications were performed using MassHunter Workstation (B.06.00 SP01, Agilent Technologies). Metabolite identifications were verified by comparison of retention times and ion fragmentation patterns with authentic standards where possible. For each fatty acid, the relative abundance of ^{13}C-labeled molecules (species containing one or more ^{13}C atoms after correction for natural abundance) was expressed as a percentage of the total of the 7 major labeled species shown.

SUPPLEMENTAL REFERENCES

Arquier, N., Vigne, P., Duplan, E., Hsu, T., Therond, P.P., Frelin, C., and D'Angelo, G. (2006). Analysis of the hypoxia-sensing pathway in Drosophila melanogaster. The Biochemical Journal *393*, 471-480.

Centanin, L., Ratcliffe, P.J., and Wappner, P. (2005). Reversion of lethality and growth defects in Fatiga oxygen-sensor mutant flies by loss of hypoxia-inducible factor-alpha/Sima. EMBO Reports *6*, 1070-1075.

Choksi, S.P., Southall, T.D., Bossing, T., Edoff, K., de Wit, E., Fischer, B.E., van Steensel, B., Micklem, G., and Brand, A.H. (2006). Prospero acts as a binary switch between self-renewal and differentiation in Drosophila neural stem cells. Developmental Cell *11*, 775-789.

Gutierrez, E., Wiggins, D., Fielding, B., and Gould, A.P. (2007). Specialized hepatocyte-like cells regulate Drosophila lipid metabolism. Nature *445*, 275-280.

Kuhnlein, R.P. (2012). Thematic review series: Lipid droplet synthesis and metabolism: from yeast to man. Lipid droplet-based storage fat metabolism in Drosophila. Journal of Lipid Research *53*, 1430-1436.

Levin, M. (2004). A novel immunohistochemical method for evaluation of antibody specificity and detection of labile targets in biological tissue. Journal of Biochemical and Biophysical Methods *58*, 85-96.

MacRae, J.I., Dixon, M.W., Dearnley, M.K., Chua, H.H., Chambers, J.M., Kenny, S., Bottova, I., Tilley, L., and McConville, M.J. (2013). Mitochondrial metabolism of sexual and asexual blood stages of the malaria parasite Plasmodium falciparum. BMC Biology *11*, 67.

Maurange, C., Cheng, L., and Gould, A.P. (2008). Temporal transcription factors and their targets schedule the end of neural proliferation in Drosophila. Cell *133*, 891-902.

McGuire, S.E., Le, P.T., Osborn, A.J., Matsumoto, K., and Davis, R.L. (2003). Spatiotemporal rescue of memory dysfunction in Drosophila. Science *302*, 1765-1768.

Owusu-Ansah, E., Yavari, A., and Banerjee, U. (2008). A protocol for in vivo detection of reactive oxygen species. Protocol Exchange.

Ryder, E., Blows, F., Ashburner, M., Bautista-Llacer, R., Coulson, D., Drummond, J., Webster, J., Gubb, D., Gunton, N., Johnson, G.*, et al*. (2004). The DrosDel collection: a set of P-element insertions for generating custom chromosomal aberrations in Drosophila melanogaster. Genetics *167*, 797-813.

Schwabe, T., Bainton, R.J., Fetter, R.D., Heberlein, U., and Gaul, U. (2005). GPCR signaling is required for blood-brain barrier formation in drosophila. Cell *123*, 133-144.

St Pierre, S.E., Ponting, L., Stefancsik, R., McQuilton, P., and FlyBase, C. (2014). FlyBase 102--advanced approaches to interrogating FlyBase. Nucleic Acids Research *42*, D780-788.

Steinhauser, M.L., Bailey, A.P., Senyo, S.E., Guillermier, C., Perlstein, T.S., Gould, A.P., Lee, R.T., and Lechene, C.P. (2012). Multi-isotope imaging mass spectrometry quantifies stem cell division and metabolism. Nature *481*, 516-519.
Xiong, W.C., Okano, H., Patel, N.H., Blendy, J.A., and Montell, C. (1994). repo encodes a glial-specific homeo domain protein required in the Drosophila nervous system. Genes & Development *8*, 981-994.
Yoshihara, M., Suzuki, K., and Kidokoro, Y. (2000). Two independent pathways mediated by cAMP and protein kinase A enhance spontaneous transmitter release at Drosophila neuromuscular junctions. Journal of Neuroscience *20*, 8315-8322.
Zhu, S., Lin, S., Kao, C.F., Awasaki, T., Chiang, A.S., and Lee, T. (2006). Gradients of the Drosophila Chinmo BTB-zinc finger protein govern neuronal temporal identity. Cell *127*, 409-422.
