# Supplementary figures and images for: Antioxidant Role for Lipid Droplets in a Stem Cell Niche of Drosophila
Source: Cell. 2015 Oct 8;163(2):340–53. doi: 10.1016/j.cell.2015.09.020 (PMC4601084; doi:10.1016/j.cell.2015.09.020)

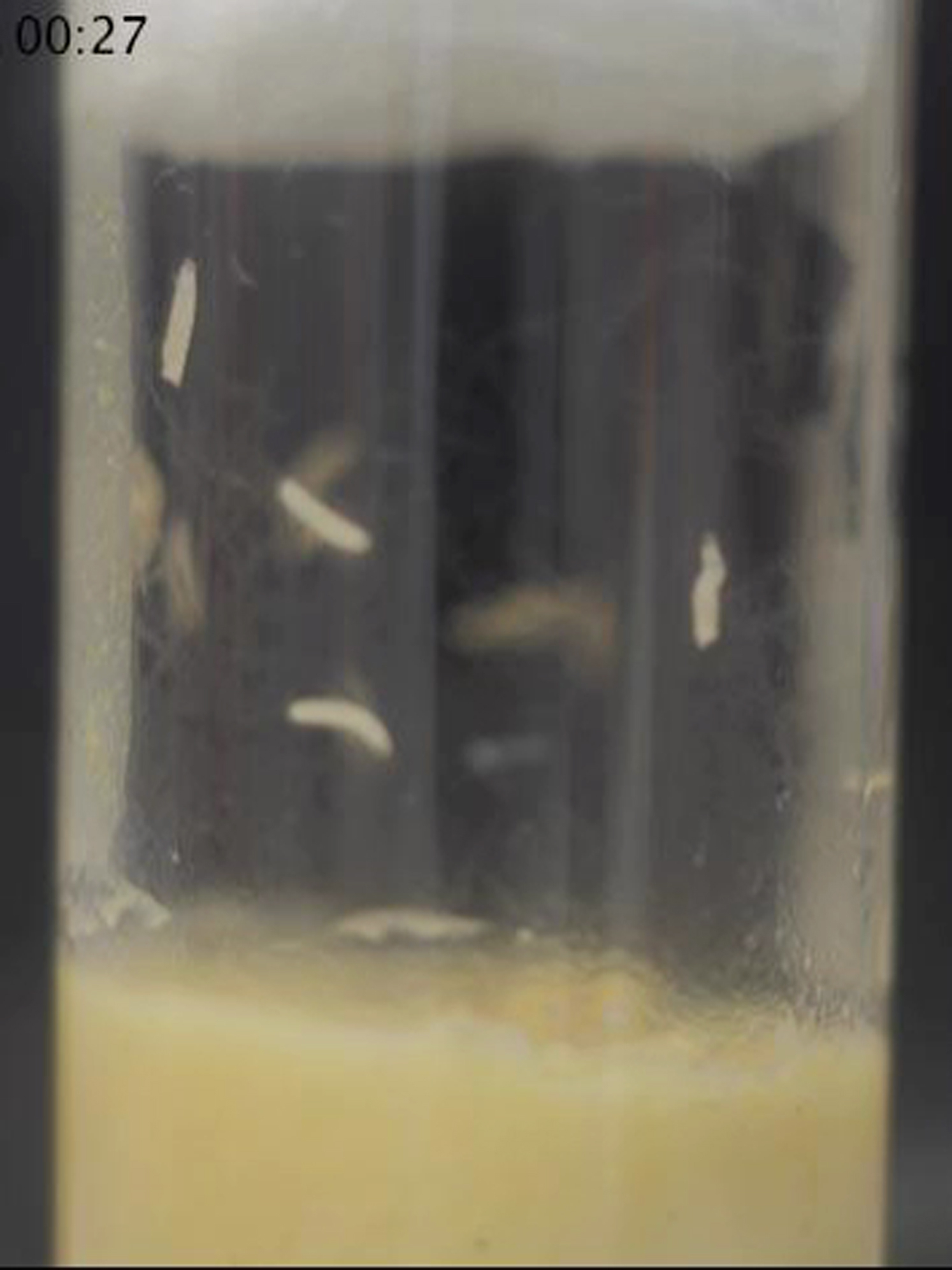

Supplement: Movie S1. Drosophila Larvae Exit the Food during Hypoxia, Related to Figure 1 — Time-lapse images of larval behavior during intermittent hypoxia were taken at 10 s intervals, and the time-stamp is shown in min. Hypoxia was progressively induced under a stream of nitrogen and larvae were rapidly returned to normoxia by removing the lid of the chamber. The timings of the cycles were hypoxia (0–6 min), normoxia (6–16 min), hypoxia (16–27 min), and then normoxia (27–42 min). [file mmc2.jpg]
